# Tuaimenal A, a Meroterpene from the Irish Deep-Sea Soft Coral *Duva florida*, Displays Inhibition of the SARS-CoV-2 3CLpro Enzyme

**DOI:** 10.1021/acs.jnatprod.2c00054

**Published:** 2022-05-12

**Authors:** Nicole E. Avalon, Jordan Nafie, Carolina De Marco Verissimo, Luke C. Warrensford, Sarah G. Dietrick, Amanda R. Pittman, Ryan M. Young, Fiona L. Kearns, Tracess Smalley, Jennifer M. Binning, John P. Dalton, Mark P. Johnson, H. Lee Woodcock, A. Louise Allcock, Bill J. Baker

**Affiliations:** † Department of Chemistry, University of South Florida, Tampa, Florida 33620, United States; § BioTools, Inc., Jupiter, Florida 33458, United States; ‡ Molecular Parasitology Laboratory (MPL), Centre for One Health and Ryan Institute, School of Natural Science, National University of Ireland Galway, H91 TK33 Galway, Republic of Ireland; ¶ School of Natural Sciences and Ryan Institute, National University of Ireland Galway, H91 TK33 Galway, Republic of Ireland; □ Department of Molecular Oncology, H. Lee Moffitt Cancer Center and Research Institute, Tampa, Florida 33612, United States

## Abstract

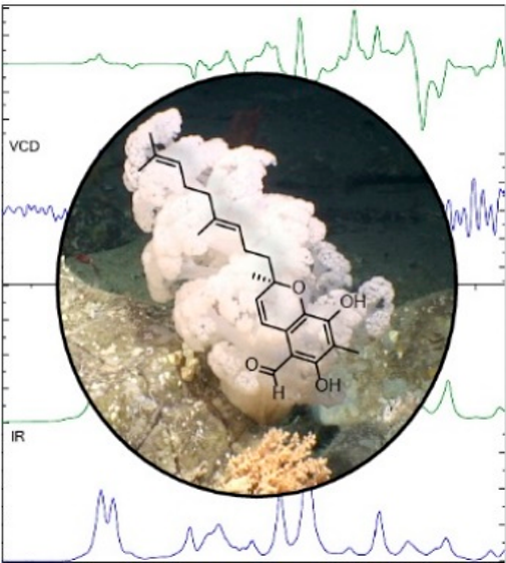

Cold water benthic environments are a prolific source of structurally diverse molecules with a range of bioactivities against human disease. Specimens of a previously chemically unexplored soft coral, *Duva florida*, were collected during a deep-sea cruise that sampled marine invertebrates along the Irish continental margin in 2018. Tuaimenal A (**1**), a cyclized merosesquiterpenoid representing a new carbon scaffold with a highly substituted chromene core, was discovered through exploration of the soft coral secondary metabolome via NMR-guided fractionation. The absolute configuration was determined through vibrational circular dichroism. Functional biochemical assays and *in silico* docking experiments found tuaimenal A selectively inhibits the viral main protease (3CLpro) of SARS-CoV-2.

Ninety-five percent of the ocean floor exceeds depths of 1000 m, where water temperatures are a constant 4 °C.^1^ Over half of the 5100 known coral species are found in the deep sea, where cold water corals create gardens in benthic regions ranging from 200 to 1000 m in depth.^1^ Utilizing both physiological and biochemical adaptations, cold water corals have adapted to survive in an environment with minimal to no light, extremely high pressures, and intense competition for resources. One biochemical adaptation to these conditions is the production of secondary metabolites.^2^ These compounds have unusual and diverse structures that confer a competitive advantage to the organisms but, incidentally, also exhibit high rates of affinity to biological targets implicated in human disease.^2^ Natural products from the deep sea constitute less than 2% of known natural products; however, the rate of bioactivity from deep-sea compounds is estimated to be as high as 75%.^2^

Across the world’s oceans, the phylum Cnidaria is second only to Porifera in the number of new natural products reported annually from invertebrates.^3^ Comprising over 3000 species, Octocorallia are a particularly rich source of natural product exploration; roughly 80% of bioactive compounds from corals have been isolated from this subclass.^4^ The Nephtheidae family comprises 20 genera and about 500 species, including *Duva florida*. Known colloquially as cauliflower corals due to their appearance, *Duva* species thrive in cold water benthic environments (Figure 1). Corals from the family Nephtheidae are known to produce steroidal and other terpenoid secondary metabolites,^4,5^ but the secondary metabolites of *D. florida* have yet to be described.

**Figure 1:**
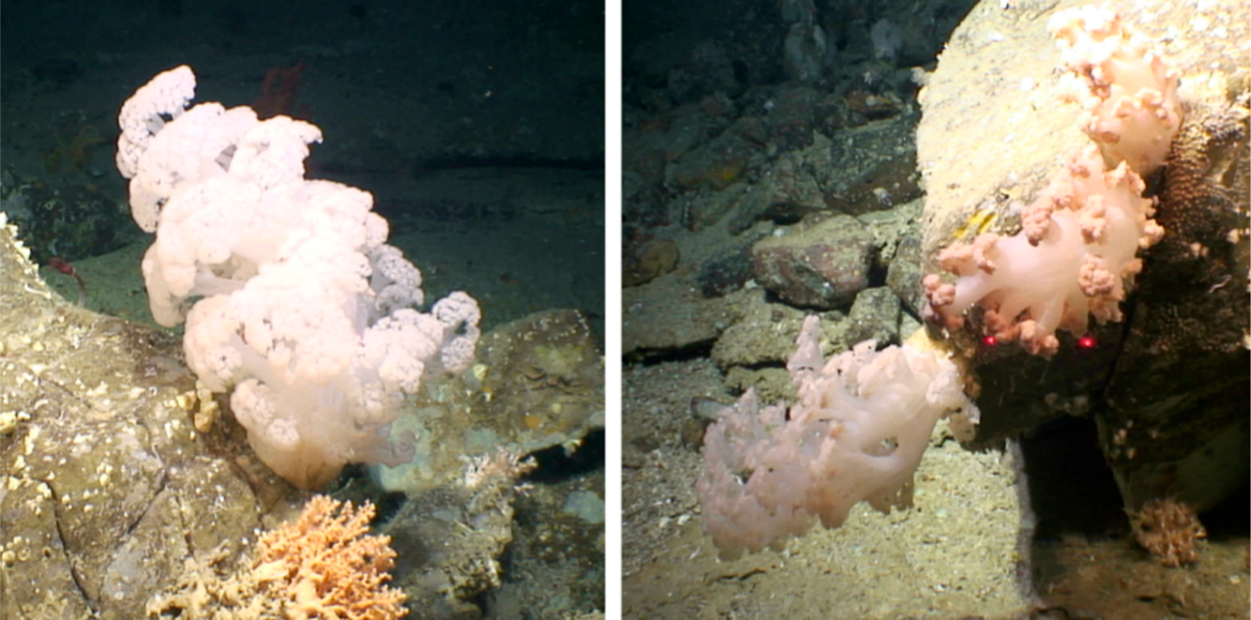
Four specimens of *Duva florida* (Cnidaria: Anthozoa: Octocorallia: Alcyonacea: Alcyoniina: Nephtheidae) were collected at a depth of 823 m from a submarine canyon north of Porcupine Bank on the Irish Continental Margin by *ROV Holland I*, deployed from *RV Celtic Explorer*. For scaling, a laser light is used (red dots indicate a span of 10 cm). Subsea photographs are copyright Marine Institute, Oranmore, Ireland. Used with permission.

In this study, we sought to analytically characterize and biochemically assess a new natural product from *D. florida* soft coral. Tuaimenal A (**1**), a compound representing a new carbon scaffold, was discovered through the exploration of the secondary metabolome of the soft coral through NMR-guided fractionation. Paired with HRMS and NMR for rigorous structure elucidation, vibrational circular dichroism (VCD) was utilized to determine the absolute configuration at the single stereogenic center. Tuaimenal A is a merosesquiterpene that possesses a highly substituted benzopyran ring system with structural similarity to tocopherol (vitamin E), although the aromatic methylation, which is typically para to the chromane oxygen, is found in the meta-position to the chromene oxygen in the structure of tuaimenal A. This subtle difference suggests a biosynthetic pathway that is divergent from that seen in the tocopherol biosynthesis, likely at the enzymatic step involving homogentisate phytyltransferase.^6^ Additionally, the oxidation pattern observed at C-6–8–8a of **1** is unusual. In fact, the majority of marine-derived meroterpenes with chromene cores, whether from corals, algae, ascidians, sponges, or microbes, often possess oxidation at the C-6 and C-8a positions and often have the aforementioned methylation, rather than an oxidized substituent at C-8. Although uncommon, oxidation at C-8 is seen in meroterpenes from the marine environment, such as scabellone A isolated from the New Zealand ascidian *Aplidium scabellum*, metachromin U from the Tasmanian sponge *Thorecta reticulata*, and chromenols isolated from the aforementioned ascidian as well as *Homeostrichus formosana*, a brown alga.^7−9^ This oxidation pattern is not commonly observed in compounds isolated from soft corals, though they are known to possess meroterpenes.^10^ Along with the aldehyde substituent on the aromatic ring, the oxidation pattern and site of methylation create the unique scaffold seen in **1**. To assess the bioactivity of this newly identified natural product, we tested its inhibitory properties in a variety of bioassays, including bacterial, fungal, and protozoan pathogens, as well as in cancer cell lines. Following *in silico* screening, tuaimenal A demonstrated inhibition of the viral main protease (3CLpro) of the severe acute respiratory syndrome coronavirus 2 (SARS-CoV-2 virus).
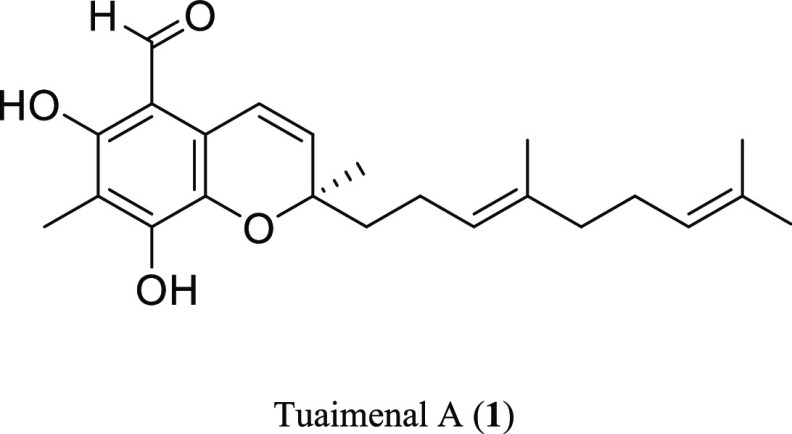


The emergence of the SARS-CoV-2 virus from Wuhan, China, in late 2019 has led to the rapid spread of the highly infectious and pathogenic virus and subsequent declaration of the COVID-19 pandemic by the World Health Organization on March 11, 2020.^11^ As of 10 May the ongoing pandemic caused by this virus has been responsible for >6 millon deaths worldwide.^12^ While vaccinations against SARS-CoV-2 are being administered globally, treatment of the disease is limited. Therefore, drug discovery and drug repurposing efforts are needed to find effective treatments for COVID-19 infections.^13,14^

Herein we report the structure and bioactivity for tuaimenal A (**1**). *In silico* docking identified the major SARS-CoV-2 proteases as targets of tuaimenal A. Biochemical assays established that tuaimenal A selectively inhibits the viral main protease (3CLpro). Therefore, tuaimenal A is a newly discovered natural product with bioactivity that may lead to novel therapeutics against SARS-CoV-2.

## Results and Discussion

### Structure Analysis of Tuaimenal A (**1**)

**Figure 2:**
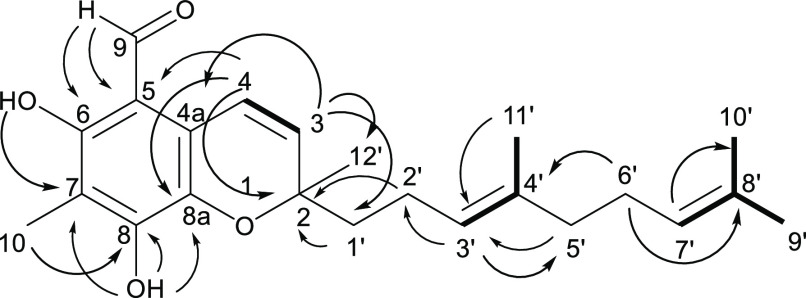
Proposed planar structure of tuaimenal A (**1**) based on NMR data. Key HMBC (**→**) and COSY (bold bonds) correlations are shown.

Tuaimenal A (**1**)^15^ was obtained as a yellow oil with a molecular formula of C_23_H_30_O_4_ based on HRESIMS data and corroborated by NMR data (Table 1). The deshielded region of the ^1^H NMR spectrum (Figure S2), informed by the phase-sensitive gHSQCAD spectrum, revealed several functional groups. The signal at δ_H_ 12.31 (OH_a_) (Figure 2) displayed no HSQC correlation to a carbon, suggesting it was on a heteroatom, and its shift was characteristic of a H-bonded phenol. The shift at δ_H_ 10.08 (H-9) correlated in the HSQC spectrum with δ_C_ 191.1 (C-9), both of which are consistent with an aldehyde function. A series of olefinic proton shifts from δ_H_ 5.07 to 6.86, taken with 12 olefinic ^13^C chemical shifts, identified a highly oxidized skeleton; the most deshielded, δ_C_ 158.2 (C-6) and 151.4 (C-8), are indicative of olefinic/aromatic oxygen-bearing carbons.

**Table 1:** NMR Spectroscopic Data (^1^H 400 MHz, ^13^C 100 MHz, CDCl_3_) for Tuaimenal A (**1**)

pos	δ_C_, type	δ_H_, mult, *J* (Hz)	HMBC
2	79.1, C		
3	132.4, CH	5.80, d (10.1)	2, 4a, 12′, 1′
4	116.4, CH	6.86, d (10.1)	2, 3, 4a, 5, 8a
4a	118.7, C		
5	107.5, C		
6	158.2, C		
7	111.7, C		
8	151.4, C		
8a	132.3, C		
9	191.1, CH	10.08, s	5, 6
10	7.6, CH_3_	2.14, s	6, 7, 8
1′	40.2, CH_2_	1.78, ov^a^ m	3, 2′, 3′
2′	22.6, CH_2_	2.12, ov m	
3′	123.3, CH	5.11, ov t	2′, 5′, 11′
4′	135.8, C		
5′	39.6, CH_2_	1.99, ov t	3′, 4′, 6′, 11′
6′	26.6, CH_2_	2.05, ov m	4′, 5′, 7′, 8′
7′	124.1, CH	5.07, ov t	10′
8′	131.4, C		
9′	25.7, CH_3_	1.68, s	7′, 8′, 10′
10′	17.7, CH_3_	1.59, s	7′, 8′, 9′
11′	16.0, CH_3_	1.58, s	5′, 3′, 4′
12′	25.4, CH_3_	1.43, s	2, 3, 1′
OH_a_		12.31, s	5, 6, 7, 8
OH_b_		6.37, s	6, 7, 8, 9

a ov: overlapping signals.

Further analysis of the 2D spectra of **1** facilitated the development of additional partial structures. The HSQC data were used to assign the remainder of the protons to their respective carbons (Table 1). Taken with the COSY spectrum, spin-coupled systems included vinylic proton δ_H_ 6.86 (H-4), which shared a COSY correlation with vinylic proton δ_H_ 5.80 (H-3). The singlet vinyl proton δ_H_ 5.11 (H-3′) shared a correlation with the 3H singlet at δ_H_ 1.58 (H_3_-11′). Additionally, δ_H_ 1.68 (H_3_-9′) showed correlations to δ_H_ 5.07 (H-7′) and 1.59 (H_3_-10′), resulting in a vinyl *gem*-dimethyl group terminating a trisubstituted olefin (Figure 2).

HMBC data were used to extend the partial structures. The signal at δ_H_ 10.08 (H-9) had multiple correlations, including δ_C_ 107.5 (C-5), 158.2 (C-6), and 111.7 (C-7), while δ_H_ 2.14 (H_3_-10) shared correlations with C-6, C-7, and δ_C_ 151.4 (C-8), forming a conjugated system consisting of two olefins and the aldehyde carbonyl (Figure 2). A proton at δ_H_ 5.80 (H-3) displayed HMBC correlations to δ_C_ 79.1 (C-2), 118.7 (C-4a), 25.4 (C-12′), and 40.2 (C-1′). Similarly, H-4 displayed HMBC correlation to C-2, δ_C_ 107.5 (C-5), and 132.3 (C-8a). Olefinic C-8a and C-4a, therefore, are in conjugation with δ_C_ 116.4 (C-4) and 132.4 (C-3). A singlet proton at δ_H_ 6.37 (OH_b_) had correlations to C-8 and C-8a, which, taken with the HMBC correlation between H-4 and C-5, establishes an aromatic ring. The C-2 shift is consistent with carbon bearing oxygen; this fully substituted carbon had correlations from H-3, H_3_-12′, and δ_H_ 1.78 (H_a_-1′).

Two partial structures established by COSY (*vide supra*) remained to be incorporated in the growing scaffold of **1**. While H_2_-1′ (δ_H_ 1.78, 2.12) and H_2_-2′ (δ_H_ 1.76, 2.13) were heavily overlapped, an HMBC correlation between those shifts and δ_C_ 79.1 (C-2) extended the scaffold. The partial structure from COSY that included δ_H_ 5.11 (H-3′) and 1.58 (H_3_-11′) could be connected to C-2′ based on an HMBC correlation between H-3′ and C-2′. The final intervention of two methylene groups at δ_C_ 39.6 (C-5′) and 26.6 (C-6′) and the terminal trisubstituted olefin was established by HMBC correlations between δ_H_ 1.99 (H-5′) and δ_C_ 123.3 (C-3′); δ_H_ 2.05 (H-6′) and δ_C_ 135.8 (C-4′) and 131.4 (C-8′); and δ_H_ 5.07 (H-7′) and δ_H_ 17.7 (C-10′), completing the linear scaffold.

Two valences remained unfilled, and the molecular formula of the established scaffold was missing one oxygen. Establishing a pyran ring between C-8a and C-2 would satisfy these last structural features. The resultant chromene is unusual in its highly substituted aromatic ring, the positions of carbon branches on the aromatic ring, and the level of oxidation on the aromatic ring.

### Evaluation of the Absolute Configuration of Tuaimenal A (**1**)

**Figure 3:**
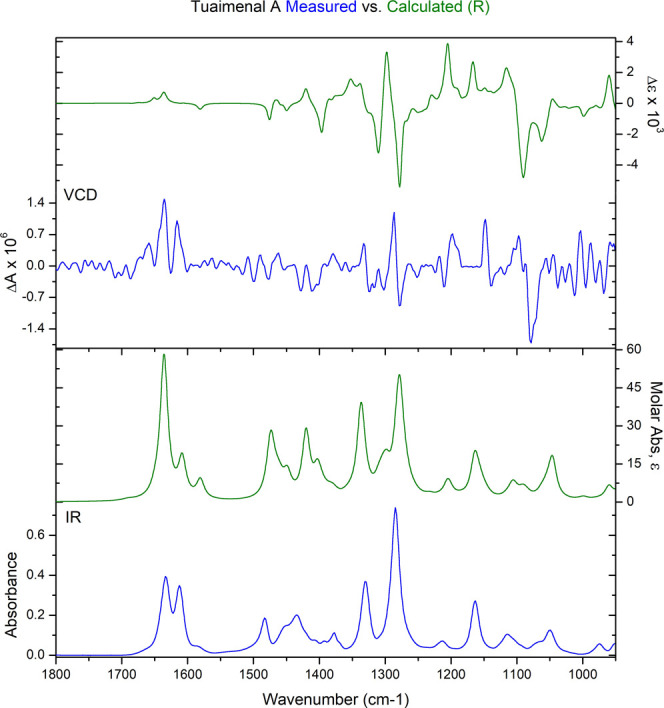
Absolute configuration at C-2 of tuaimenal A (**1**) was determined to be the *R* configuration based on VCD analysis. Top graph shows the congruence between the calculated VCD for the *R*-configured stereocenter (green) and the measured VCD (blue). In the lower graph, the calculated FTIR absorbance (green) also demonstrates congruence with the calculated data (blue).

Determination of the absolute configuration of **1** was achieved using VCD, a method that can be employed directly on chiral molecules in solution phase.^16−19^ The flexible hydrocarbon tail gave rise to a large number of low-energy conformations, which presented a challenge for density functional theory (DFT) calculations. A truncated version of the molecule was initially studied substituting an ethyl group for the flexible hydrocarbon tail. This had a reduced number of low-energy conformers (nine), which were rapidly calculated for comparison to the experimental spectra. While there was some congruence, the overall comparison was not satisfactory. A thorough molecular mechanics search of tuaimenal A yielded over 800 conformers in a 5 kcal/mol range. Using a small Linux cluster with 64 available cores, DFT calculations were performed on all of the conformers at the B3LYP/6-31G(d) level.^20^ After removing duplicates and higher energy structures, the resulting 338 unique conformers were Boltzmann averaged to produce the final theoretical spectrum (Figure 3). The *R* enantiomer was used for the calculation, and a match to the measured spectrum confirmed the configuration of tuaimenal A as *R*. The comparison of experimental and theoretical spectra was quantified^21,22^ using BioTools CompareVOA software, with high neighborhood similarity for IR (90.6) and VCD (57.5), enantiomeric similarity index for VCD (35.6), and a confidence level of 86%. A fairly weak VCD signal gave rise to some noise in the experimental spectrum, which likely reduced the confidence level slightly. Visual comparison of the data makes clear that the assignment is correct, with 11 of the most intense VCD bands well correlated to the experimental data. Overall, this proved to be an effective method to determine the absolute configuration of tuaimenal A.

### Rigid Docking of Tuaimenal A (**1**) in SARS-CoV-2 Protein Targets

Four protein targets were selected for *in silico* experiments based on their critical roles in SARS-CoV-2 infections. The main protease, also known as 3CLpro or Mpro, is the viral protease responsible for cleaving 12 nonstructural proteins (Nsp4–Nsp16), allowing for viral maturation.^23^ Inhibition of this protease prevents viral replication, making it a highly attractive drug target.^24,25^ Similarly, the papain-like protease (PLpro) assists with viral replication by cleaving three nonstructural proteins (Nsp1–Nsp3).^23^ The host transmembrane serine protease 2 (TMPRSS2) assists in the activation and proliferation roles for SARS-CoV-2, and inhibition of this protein can block cell entry.^26^ RNA-dependent RNA-polymerase (RdRp) is a critical component for both replication and transcription of this positive-strand RNA virus and, therefore, another important protein to target.^27^

For docking experiments, Schrödinger’s Glide XP scoring function was utilized for ranking and analyzing poses of **1**. Glide scores are reported for each pose and represent a correlation between the protein–ligand complex and the binding energy.^28^ The criterion for selecting favorable poses was a threshold of −7.0 or lower, and ligands are considered to have favorable binding interactions with the target protein when presenting these scores. In the rigid docking, **1** showed the most promising profile for binding to 3CLpro (Figure S12), with a lowest Glide score of −8.925. The favorable binding is attributed to pi–pi stacking interactions with His41 and various hydrogen bonds (Figure S13). Docking tuaimenal A into PLpro, TMPRSS2, and RdRp resulted in a lowest Glide score of −8.533, −8.282, and −7.419, respectively. Rigid docking results provided a basis for the *in silico* experiments of tuaimenal A, and flexible docking was conducted to further investigate tuaimenal A affinity for the various SARS-CoV-2 protein targets.

### Flexible Docking of Tuaimenal A (**1**) in SARS-CoV-2 Protein Targets

A novel CHARMM-based flexible docking protocol, CIFDock,^29^ was employed to dock **1** into the same four protein targets (3CLpro, PLpro, RdRp, and human TMPRSS2). This method allows full flexibility of the target receptor and ligand, providing a more thorough conformational space search of tuaimenal A in the active sites of these targets. Rigid docking of tuaimenal A suggested 3CLpro was the most promising of the protein targets. After using CIFDock to flexibly dock tuaimenal A into these proteins, final generated poses were assigned a Glide score using the Glide XP scoring function. The average Glide scores for each tuaimenal A pose in 3CLpro was −10.77 with a lowest scoring pose of −12.42. Docking tuaimenal A with the remaining three proteins, PLpro, RdRp, and human TMPRSS2, resulted in an average Glide score of −7.14, −7.00, and −7.26, respectively. The results from the flexible docking then suggest that tuaimenal A would bind favorably to 3CLpro and may potentially bind to PLpro, RdRp, and TMPRSS2 (Table 2, Figures S9–S11). Low binding potentials for RdRp are likely attributed to the fact that RdRp is generally inhibited through covalent rather than intermolecular interactions.^27^

**Table 2:** Results from the Flexible Docking of Tuaimenal A (**1**) into the Four SARS-CoV-2 Protein Targets^a^

protein target	average score	lowest score
3CLpro	–10.77	–12.42
PLpro	–7.14	–8.28
TMPRSS2	–7.26	–8.64
RdRp	–7.00	–7.39

a Glide scores are averaged across three protein conformations for 3CLpro and PLpro and two for TMPRSS2 and RdRp.

**Figure 4:**
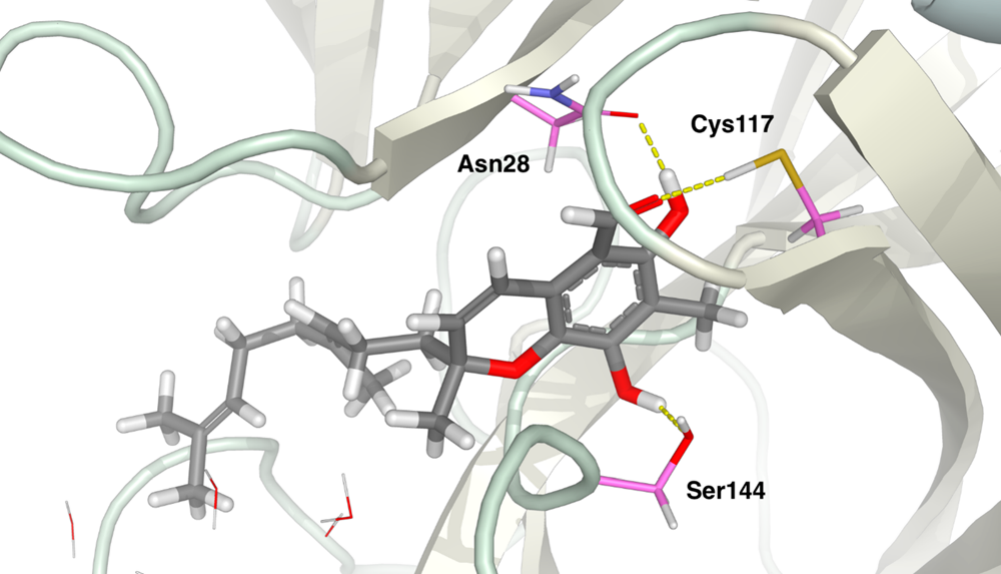
Final pose of the flexible docking of tuaimenal A (**1**) into the main protease (3CLpro). Hydrogen bonds (in dashed yellow lines) can be seen between the ligand and side chains Asn28, Cys117, and Ser144.

The protease–ligand interactions of the lowest scoring final pose between 3CLpro and tuaimenal A (**1**) were visualized (Figure 4). Multiple hydrogen-bonding interactions can be seen due to the many acceptor/donor groups on the topology of **1**. Both hydroxy groups form hydrogen bonds with the protein: one hydroxy group with Asn28 and the other with Ser144 and Gly146. The hydrogen bond between **1** and Gly146 is to a backbone atom and is thus not pictured in Figure 4. The tuaimenal A aldehyde oxygen forms hydrogen bonds with Cys117 and Asn119. Finally, the ring oxygen forms a hydrogen bond with the backbone nitrogen of Cys145. Overall, **1** forms a network of hydrogen bonds with the 3CLpro that results in an intensely favorable binding with this SARS-CoV-2 protease target. Although the active site of Mpro is known to have hydrophobic pockets,^24^ the terpene tail of tuaimenal A does not appear to occupy these sites in the final docked pose. The terpene tail extends beyond a hydrophobic pocket and into a more polar region of the protein.

### Inhibitory Activity of Tuaimenal A (**1**) against the 3CLpro

**Figure 5:**
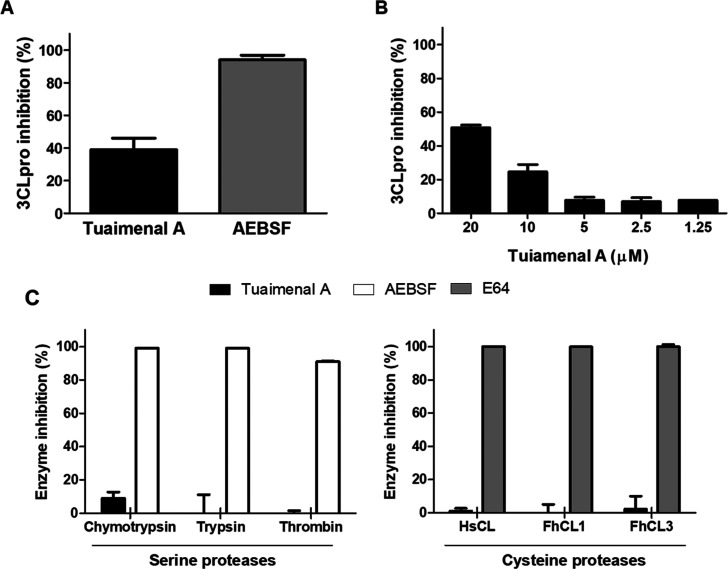
(A, B) Inhibition of 3CLpro activity and evaluation of the specificity of tuaimenal A (**1**) as a protease inhibitor. (A) The activity of 3CLpro (500 nM) was measured in the presence of tuaimenal A (**1**) (20 μM) or the broad-spectrum serine protease inhibitor AEBSF (5 mM). (B) Determination of the IC_50_ of **1** against 3CLpro. The activity of 3CLpro (500 nM) was assayed in the presence of a range of **1** concentrations (1.25 to 20 μM). Inhibition is presented relative to the activity of 3CLpro in the absence of inhibitors, and error bars indicate standard deviation of three separate experiments. (C) The ability of **1** to inhibit different proteases was evaluated using a panel of serine and cysteine proteases. The activity of the human proteases chymotrypsin (4.0 nM), trypsin (168 nM), thrombin (800 pM), and HsCL (0.2 nM) or of the unrelated parasite proteases FhCL1 (2.7 nM) and FhCL3 (5 nM) was tested in the presence of 20 μM **1** (dark bars). The broad-spectrum serine proteases inhibitor AEBSF (5 mM; white bars) or cysteine proteases inhibitor E64 (100 μM, gray bars) was used as a positive control. Inhibition is presented relative to the activity of each enzyme in the absence of inhibitors, and error bars indicate standard deviation of three separate experiments.

To validate the *in silico* docking experiments, the enzymatic activity of recombinant 3CLpro was measured in the presence or absence of **1**. Initially, the compound was screened against the 3CLpro at 20 μM. Tuaimenal A inhibited ∼40% of the 3CLpro activity, compared to ∼100% inhibition obtained with 5 mM AEBSF, a broad-spectrum serine protease inhibitor (Figure 5A). We proceeded to assess the IC_50_ of tuaimenal A against 3CLpro; our data showed that 50% of the activity of the 3CLpro can be abrogated by tuaimenal A at 21 μM (Figure 5B). Moreover, further analyses revealed that tuaimenal A behaves as a specific 3CLpro inhibitor, as complementary assays with a range of serine and cysteine proteases showed no inhibition when tuaimenal A was used at the same concentration of 20 μM (Figure 5C).

## Conclusions

Four specimens of the Irish deep-sea coral *D. florida* were explored utilizing NMR-guided fractionation methods. The chemistry of the family Nephtheidae to which it belongs has been previously described; however, this is the first analysis of *D. florida*. A new natural product, tuaimenal A (**1**), was discovered. This meroterpene represents a new carbon scaffold, a highly substituted merosesquiterpenoid chromene. The benzopyran ring system includes an aldehyde, two phenol features, and a methyl group.

Tuaimenal A (**1**) inhibits the main protease for SARS-CoV-2. *In silico* docking experiments identified 3CLpro as a highly favorable target for tuaimenal A (**1**). Biochemical studies further confirmed that tuaimenal A effectively and selectively inhibits the main protease of SARS-CoV-2. Interestingly, several natural products from terrestrial sources that share a chromene core demonstrate antiviral activity ascribed to 3CLpro inhibition.^30−33^ Many drug discovery campaigns have sought to identify inhibitors against the main protease of both SARS-CoV and SARS-CoV-2, highlighting it as a prime antiviral target. Though previous inhibitors have been described, most have been unsuccessful in making their way to the clinic.^33−35^ Recently, Pfizer’s COVID-19 oral antiviral drug targeting 3CLpro, Paxlovid, was shown to reduce the risk of hospitalization or death by 89% and has received Emergency Use Authorization by the U.S. Food and Drug Administration.^36,37^ Despite this success, we still have a very limited number of drugs to treat COVID-19, including remdesivir and monoclonal antibody therapies. To ensure we are well prepared for new variants of concern, especially those that may confer resistance to current prophylactic or therapeutic treatment options, we need to develop new therapeutics to treat COVID-19 infections. The discovery of tuaimenal A as an inhibitor of SARS-CoV-2 3CLpro suggests that further *in vitro* and *in vivo* studies with this compound and/or derivates of it could culminate in the development of a novel COVID-19 drug. Further, cell-based studies have shown that tuaimenal A has low to no toxicity in cervical cancer cells, which further supports it specificity toward SARS-CoV-2 3CLpro, as these cells are sensitive to other protease inhibitors.^38,39^ The discovery of tuaimenal A points to the importance of chemical explorations of deep-sea organisms. Careful sampling and analysis in cold water coral systems can promote drug discovery efforts and underscore the importance of conserving of these natural resources.

## Experimental Section

### General Experimental Procedures

The optical rotation was measured using a Rudolph Research Analytical AUTOPOL IV digital polarimeter. UV absorptions were measured using an Agilent Cary 60 UV–vis spectrophotometer. The IR spectrum was recorded with an Agilent Cary 630 FTIR spectrometer and a PerkinElmer Spectrum Two equipped with a UATR (single reflection diamond) sample introduction system. All ^1^H NMR spectra were recorded at 298 K on a Bruker Neo 400 MHz or on a Varian 500 MHz Direct Drive instrument with direct detection, and ^13^C NMR spectra were recorded at 100 or 125 MHz, respectively. Chemical shifts are reported with the residual chloroform (δ_H_ 7.27; δ_C_ 77.0) and methanol (δ_H_ 3.31; δ_C_ 49.1) signals as internal standards for ^1^H and ^13^C spectra. Commercial silica gel 230–400 mesh was used to load samples for MPLC. MPLC was performed on a Teledyne Isco CombiFlash Rf200i with UV detection and a RediSep RF silica flash column. Semipreparative normal phase HPLC was performed on a Shimadzu LC-20 AT system with an evaporative light scattering detector and UV detection using semipreparative (Phenomenex Luna silica; 250 × 10 mm, 5 μm) or analytical (Phenomenex Luna C18; 250 × 4.6 mm, 5 μm) conditions. Semipreparative nonaqueous reversed phase was performed on a Shimadzu LC20-AT system with a photodiode array detector using semipreparative (Phenomenex Luna C18; 250 × 10 mm, 5 μm) conditions. All solvents were HPLC grade (>99% purity) unless stated otherwise and were obtained from Fisher Scientific. HRESIMS experiments were performed using an Agilent 6230 TOF LC-MS.

### Collection of *Duva florida* Samples

The four individuals of *D. florida* were collected by *ROV Holland 1* from the deep sea (54.26007932 N, 11.58046619 W) at a depth of 823 m during a 2018 cruise of *RV Celtic Explorer* (Cruise CE18012). *In situ* photographs were taken, and the specimens subsequently identified as *Duva florida* based on polyp ramification, size, and number. Any epibionts were removed, and a small voucher was removed and placed in 96% EtOH. From this voucher, a short region of the mitochondrial genome was sequenced (GenBank Accession No. ON127699) and compared to unpublished sequences of *D. florida* and other closely related nephtheid species to confirm the identification. The remainder of the biomass was frozen at −80 °C on board the *RV Celtic Explorer*. The samples were lyophilized upon arrival at National University of Ireland, Galway, and stored at −20 °C, then transported to University of South Florida and stored at −20 °C until further processing.

### Extraction and Isolation of Natural Products

Upon arrival at the University of South Florida, the specimens were extracted with 100% CH_2_Cl_2_ via reflux (40 °C) with a Soxhlet apparatus. From the 88 g of lyophilized sample, 14.3 g of extract was obtained. A dichloromethane/water partition was performed, resulting in 13.7 g of extract in the organic layer. Initial separation was performed using NP MPLC with a silica column. A linear gradient of hexanes to EtOAc over 30 min, followed by isocratic 100% EtOAc for 8 min and then 20% MeOH and 80% EtOAc for 7 min, was used. All resulting fractions were dried using passive air or nitrogen. Thirteen fractions were obtained, and based on NMR spectroscopic data, the fourth through sixth fractions (eluting roughly around 0% to 35% EtOAc) were selected for further purification. Iterations of NP HPLC were performed leading to the isolation of 36.8 mg of tuaimenal A (**1**).

#### Tuaimenal A (**1**):

yellow oil; [α]^20^_D_ 0.7 (*c* 0.007, CHCl_3_); UV (MeCN) λ_max_ (log ε) 307 nm (1.5) with additional peaks noted at λ (log ε) 258 (1.4) and 369 nm (0.3); IR ν (thin film) 2980, 2930, 2880, 1633, 1612, 1483, 1329, 1284 cm^–1^; ^1^H and ^13^C NMR data, Table 1; HRESIMS *m*/z 371.2216 [M + H]^+^ (calcd for 371.2217, C_23_H_31_O_4_).

### Vibrational Circular Dichroism Measurements

Tuaimenal A (**1**) (5.7 mg) was dissolved in 220 μL of CDCl_3_ and transferred to a BaF_2_ IR cell with a path length of 100 μm. Instrumentation was a BioTools ChiralIR 2X DualPEM FT-VCD, with a resolution of 4 cm^–1^ and PEM maximum frequency of 1400 cm^–1^. The sample was measured for 24 blocks of 1 h each while purged with dry air to remove water vapor. The IR was processed by solvent subtraction and offset to zero at 2000 cm^–1^. The VCD blocks were averaged, then subtracted using a solvent baseline to produce the final spectrum.

### Vibrational Circular Dichroism Calculations

(*R*)-Tuaimenal A (**1**) was subjected to a GMMX (MMF94) search using BioTools ComputeVOA software to find the lowest energy conformers in a 5 kcal/mol range. A total of 805 conformers were minimized using Gaussian 09 at the 631G(d)/B3LYP level with the CPCM solvent (CHCl_3_) model. IR and VCD frequencies were calculated at the same level, then duplicates were removed. The 338 lowest energy unique conformers were then Boltzmann averaged and plotted with a line width of 5 cm^–1^. IR and VCD spectra were then frequency scaled by a factor of 0.968 and compared to the experimental data.

### Ligand Preparation

The tuaimenal A structure was prepared for molecular docking studies with Schrödinger’s Ligand Preparation Tool (LigPrep).^40^ Protonation states of **1** were generated using Epik^41^ around a target pH of 7.0 ± 2.0. The structures were able to tautomerize and were desalted before final poses were given. The specified chirality of the molecule was retained. The generated ligands for **1** were capped at 32 conformations of energetically minimized output structures.

### Protein Structure Preparation and Grid Generation

Structures of SARS-CoV-2 protein targets considered in this work were largely collected from the PDB: 3CLpro/Mpro (6LU7),^42^ PLpro (6W9C),^43^ and RdRp (7BV2),^27^ the exception being TMPRSS2, which was built as a homology model using SWISS-MODEL.^44^ To relax these protein structures under biologically relevant conditions, structures of 3CLpro, PLpro, RdRp, and TMPRSS2 were solvated and neutralized in water boxes and then subjected to molecular dynamics simulations according to the following procedures. First, all receptor targets were preprocessed through CHARMMing.org(45) (CHARMM web interface and graphics) to determine amino acid protonation states (via PROPKA),^46^ add missing hydrogens, and assign correct bond orders. Each protein was then solvated with 46 656 TIP3 waters in a 90 × 90 × 90 Å^3^ water box using CHARMM (Chemistry at Harvard Molecular Mechanics)^47^ version C41A1 with C36 protein parameters. The number of counterions needed to achieve a net zero charge for the system was calculated and randomly placed in the water box using a Monte Carlo-based method. Next, each system was heated from 110 to 310 K using CHARMM molecular dynamics over a 400 ps time scale. To ensure each protein was then sufficiently relaxed, each system was then equilibrated for another 20 ns at 310 K. From this equilibrium trajectory, unique conformations of each protein structure were selected by clustering the RMSD of each active site. Active site definitions for each protein can be found in the Supporting Information (Table S1). Using an RMSD threshold of 2 Å, an average of seven unique protein conformations were generated for each SARS-CoV-2 protein target to be used in the docking procedures. As a result of the increased computational time of flexible docking relative to rigid docking, three protein conformations of 3CLpro and PLpro (and two conformations of TMPRSS2 and RdRp) were used as receptors for the flexible docking in this study.

Final protein conformations generated from a flexible docking of antiviral compounds into these prepared protein systems (from another study) were also used as initial target receptors for the rigid docking portion of this current study. These final structures have already adopted favorable conformations to accommodate large antiviral ligands through application of the same flexible ligand/flexible receptor docking protocol used in this work (CIFDock). In total, five “optimized” final protein conformations were used as different initial receptor structures for all protein targets in the rigid docking portion of this study (i.e., tuaimenal A (**1**) was rigidly docked into five different conformers each of 3CLpro, PLpro, TMPRSS2, and RdRp).

Schrödinger’s Protein Preparation Wizard^48^ was used in convert clustered structures resulting from molecular dynamics simulations in CHARMM to receptor structure files compatible with Schrödinger’s Glide Docking.^49^ Correct bond orders were assigned, missing hydrogens were added, disulfide bonds were created, waters beyond 5 Å from heterogroups were deleted, and protonation states were generated using PropKa for a pH range of 7.0 ± 2.0. After preprocessing was complete, water orientations were sampled and then optimized. Waters with less than three hydrogen bonds to non-hydrogen atoms were removed from the protein structure. A restrained minimization was then performed in the final processing step, where heavy atoms were converged to an RMSD of 0.30 Å using an OPLS3e force field. The Glide Receptor Grid Generation tool was used to convert complete protein structure files into minimized receptor structure files represented as simplified interaction grids. For the optimized protease conformations, binding residues were specified, and the centroid of the selected residues served as the center of the binding site. Information on the residue numbers selected in the definition of the active site of each protein target can be found in the Supporting Information (Table S1). After selecting the binding site, rotatable groups were visually selected based on proximity to the centroid and with careful consideration of any residue that would have obstructed the active site. Rotatable groups were selected separately for each conformation following these guidelines.

### Flexible Docking

The CHARMM-based flexible docking method (CIFDock)^29^ was used to flexibly dock (flexible ligand/flexible receptor) tuaimenal A (**1**) into all protein targets (e.g., 3CLpro, PLpro, TMPRSS2, and RdRp). CIFDock incorporates induced fit, in which ligand and protein conformational degrees of freedom can affect one another during the initial approach and complexation. CIFDock thus allows full flexibility of the protein binding site and the ligand, as well as retaining explicit solvent interactions throughout the docking procedure. To achieve this level of flexibility, bulky residues in the active site of a protein that may intrude upon the user-defined binding site when sampled with dynamics were first mutated to alanine. This allows for a more “open” binding site that is better able to accommodate larger and more flexible ligands. Ligand flexibility is achieved by generating conformations using the Confab module of Open Babel.^50^ A total of 200 ligand conformations were generated via Confab by rotating any rotatable bonds in the molecule and saving these conformations as unique structure files. Next, these unique ligand conformations were randomly placed in the protein active site and 5 ps of SGLD (Self-Guided Langevin Dynamics)^51^ was run on the ligand with a fixed receptor to produce a variety of initial conformations. Finally, those residues that were mutated to alanine in the user-defined binding site are back-mutated to their original residues. Finally, the entire ligand and surrounding protein residues in the binding site (up to 9 Å outward from any ligand atom) are simulated with two rounds of 2 ps of SGLD (once before explicit waters are added back in and once after) to allow sampling of the protein–ligand complex conformational space, with possible ligand-induced conformational changes of the active site. A set of custom-designed scoring functions are combined into an ensemble docking score to evaluate the poses generated by CIFDock by analyzing interaction energies (e.g., electrostatic interactions, van der Waals, solvation energies). The top 20 ranked poses, as determined by the CIFDock scoring functions, were then also given a Glide score by evaluating the poses with Schrödinger’s Glide XP scoring function.

### Screening SARS-CoV-2 Inhibitory Properties of Tuaimenal A (**1**)

#### Enzymes

The recombinant *Fasciola hepatica* cathepsin L1 (FhCL1) and L3 (FhCL3) zymogens were produced in methanotrophic yeast *Pichia pastoris* and purified as previously described.^52^ For the enzymatic assays, the recombinant *F. hepatica* zymogens were activated by mixing each of them with activation buffer (0.1 M sodium citrate buffer, 2 mM DTT, 2.5 mM EDTA, pH 4.5) and incubating for 2 h (FhCL1) or 3 h (FhCL3) at 37 °C. The human cathepsin L (HsCL) was activated as described above for 45 min at 37 °C. HsCL, chymotrypsin, trypsin, and thrombin were acquired from Sigma-Aldrich.

The SARS-CoV-2 protease, 3CLpro, sequence was codon optimized for expression in *Escherichia coli* cells and synthesized in the pET-28a(+) vector (kanamycin resistant) with a C-terminal His-tag (Genscript). The synthesized vector was transformed into BL21 competent *E. coli* cells (ThermoFisher Scientific), which were cultured in LB broth containing kanamycin (1 μg/mL) at 37 °C. Once an OD_600_ was reached, protein expression was induced with 1 mM isopropyl-β-d-1-thiogalactopyranoside (IPTG; ThermoFisher Scientific) for 3 h at 30 °C. Following centrifugation at 10000*g* for 10 min at 4 °C, the bacteria pellet was digested with lysozyme (10 μg/mL) and sonicated. Subsequent centrifugation at 10000*g* for 10 min at 4 °C was used to recover the soluble recombinant 3CLpro within the supernatant that was purified using the Profinia Affinity Chromatography Protein Purification System (Bio-Rad), with the mini Profinity IMAC and mini Bio-Gel P-6 desalting cartridges (Bio-Rad).

#### Assay Conditions

Unless otherwise stated, all enzymes were assayed in a 100 μL reaction volume using appropriate buffer (Table 3) for each enzyme. Enzyme concentration and substrates used in the screening assays are presented in Table 3. Initially, the reaction buffer was mixed with 20 μM tuaimenal A (**1**) (for IC_50_ experiments serial dilutions from 20 μM were used), and the enzyme was then added to the reaction and incubated for 15 min at 37 °C before the fluorogenic substrate was added. The broad-spectrum inhibitors E-64 (100 μM; Sigma-Aldrich) and AEBSF (5 mM; Sigma-Aldrich) were used as a positive control inhibitor of cysteine and serine proteases, respectively. Hydrolytic activity was measured over 1 h at 37 °C as relative fluorescent units (RFU) in a PolarStar Omega spectrophotometer (BMG LabTech). All assays were carried out in triplicate, and the results were analyzed using GraphPad Prism 5.0 software.

**Table 3:** Assay Conditions for Each Enzyme Screened with Tuaimenal A (**1**)

enzyme (concentration)	buffer	substrate
HsCL (0.2 nM)	sodium acetate^a^	Z-Phe-Arg-NHMec (20 μM)
bovine α-chymotrypsin (4 nM)	Tris HCl^b^	Suc-Ala-Ala-Pro-Phe-NHMec (20 μM)
bovine trypsin (168 nM)	Tris HCl	Z-Leu-Arg-NHMec (20 μM)
bovine thrombin (800 pM)	Tris HCl	Z-Gly-Pro-Arg-NHMec (20 μM)
3CLpro (500 nM)	Hepes buffer^c^	LGSAVLQ-rhodamine 110-dp (20 μM)
FhCL1 (2.7 nM)	Tris HCl	Z-Leu-Arg-NHMec (20 μM)
FhCL3 (5 nM)	sodium acetate	Z-Gly-Pro-Arg-NHMec (20 μM)

a 100 nM sodium acetate, 1 mM EDTA, 1 mM DTT, 0.01% Brij L23, pH 5.5.

b 50 mM Tris, 100 mM NaCl, 1 mM EDTA, pH 7.0.

c 20 mM Hepes, 2 mM EDTA, pH 7.4.

The fluorogenic substrates were acquired from Bachem or BostonBiochem (LGSAVLQ-rhodamine 110-dp).

## Supporting Information Available

^1^H, ^13^C, 2D NMR, HRESIMS, UV, FTIR spectra, and VCD data for tuaimenal A (**1**); binding site residues for rigid docking with 3CLpro, Plpro, RdRp, and TMPRSS2 protein targets (PDF)
