# Supplementary material for: Tuaimenal A, a Meroterpene from the Irish Deep-Sea Soft Coral Duva florida, Displays Inhibition of the SARS-CoV-2 3CLpro Enzyme
Source: J Nat Prod. 2022 May 12;85(5):1315–23. doi: 10.1021/acs.jnatprod.2c00054 (PMC9127705; doi:10.1021/acs.jnatprod.2c00054)
Supplement: Supplementary file 1 — np2c00054_si_001.pdf [file np2c00054_si_001.pdf]

Supplemental Information for:

Tuaimenal A, a Meroterpene from the Irish Deep-Sea Soft Coral *Duva florida*, Displays Inhibition of the SARS-CoV-2 3CLpro Enzyme.

Nicole E. Avalon,[†] *Jordan Nafie,*[§] *Carolina De Marco Verissimo,*[‡] *Luke C. Warrensford,*[†] *Sarah G. Dietrick,*[†] *Amanda R. Pittman,*[†] *Ryan M. Young,*[††] *Fiona L. Kearns,*[†] *Tracess Smalley,*[‡‡] *Jennifer M. Binning,*[‡‡] *John P. Dalton*[‡] *Mark P. Johnson,*[††] *H. Lee Woodcock,*[†] *A. Louise Allcock,*[*,††] *and Bill J. Baker*[*,†]

Contact information for the Corresponding Authors: louise.allcock@nuigalway.ie (ALA), bjbaker@usf.edu (BJB)

[†]Department of Chemistry, University of South Florida, Tampa, FL, USA. [§]BioTools, Inc., Jupiter, FL, USA. [‡]Molecular Parasitology Laboratory (MPL), Centre for One Health and Ryan Institute, School of Natural Science, National University of Ireland Galway, Galway, Republic of Ireland. [††]School of Natural Sciences and Ryan Institute, National University of Ireland Galway, H91 TK33 Galway, Republic of Ireland. [‡‡]Department of Molecular Oncology, H. Lee Moffitt Cancer Center and Research Institute; Tampa, FL, USA.

	Page
Figure S1: Tuaimenal A (**1**) ESI-HRMS.	2
Figure S2: Tuaimenal A (**1**) ^{1}H NMR spectrum (400 MHz, $CDCl_3$).	2
Figure S3: Tuaimenal A (**1**) ^{13}C NMR spectrum (100 MHz, $CDCl_3$).	3
Figure S4: Tuaimenal A (**1**) gCOSY spectrum (400 MHz, $CDCl_3$).	3
Figure S5: Tuaimenal A (**1**) HSQC spectrum (400 MHz, $CDCl_3$).	4
Figure S6: Tuaimenal A (**1**) HMBC spectrum (400 MHz, $CDCl_3$).	4
Figure S7: Tuaimenal A (**1**) UV spectrum in ACN (10 μg/mL).	5
Figure S8: Tuaimenal A (**1**) FTIR spectrum, thin film.	5
Figures S9: Final pose from the flexible docking of tuaimenal A (**1**) into PLpro.	6
Figure S10: Final pose from the flexible docking of tuaimenal A (**1**) into TMPRSS2.	6
Figure S11: Final pose from the flexible docking of tuaimenal (**1**) A into RdRp.	7

Figure S12: Glide scores for SARS-CoV-2 proteins targets and tuaimenal A (**1**). 8
Figure S13: Final pose of tuaimenal A (1) Glide docking with 3CLpro. 8
Figure S14: Structure of Mpro with the final ligand poses. 9
Rigid Docking Methods and Results 9
Table S1: Residue numbers selected to define the active site of each protein target. 10
Table S2: Glide scores received by tuaimenal A (**1**) against protease target poses. 11
Table S3: Absolute Configuration Determination Report. 12-19

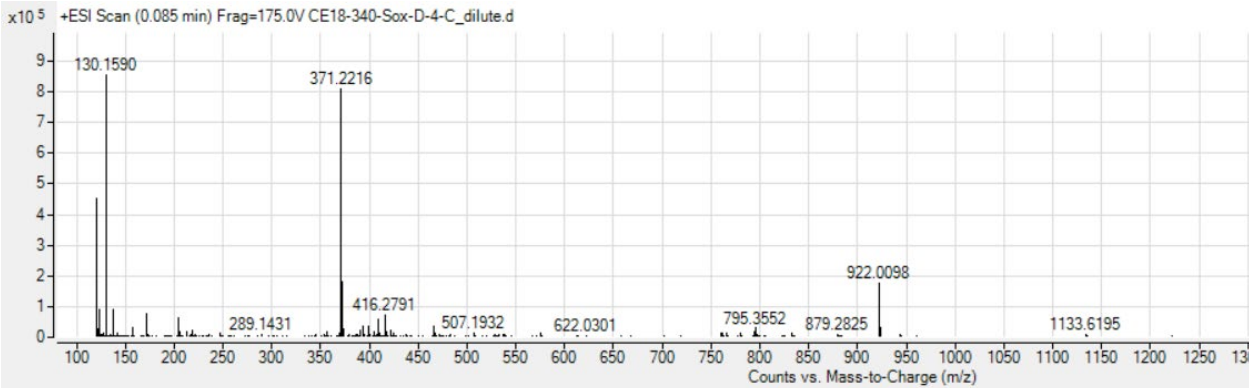

Figure S1: Tuaimenal A (**1**) ESI-HRMS. The base peak of 371.2216 is consistent with the $[M + H]^+$ (calculated for $C_{23}H_{31}O_4$ 371.2217, Δmmu 0.1).

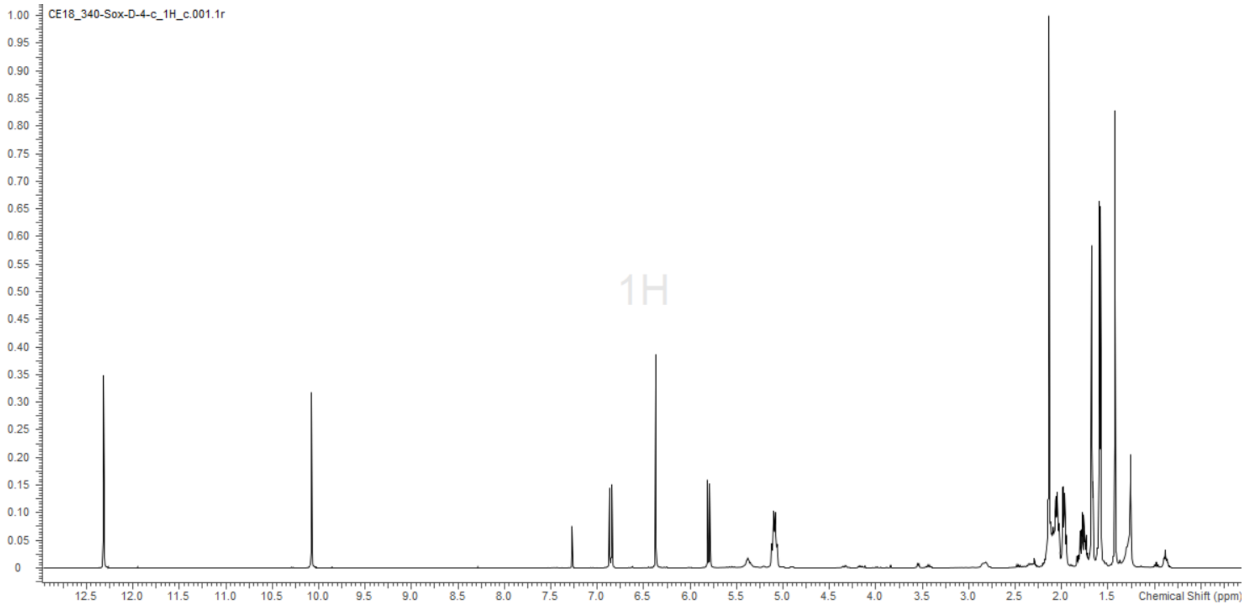

Figure S2: Tuaimenal A (**1**) ^{1}H NMR spectrum (400 MHz, $CDCl_3$).

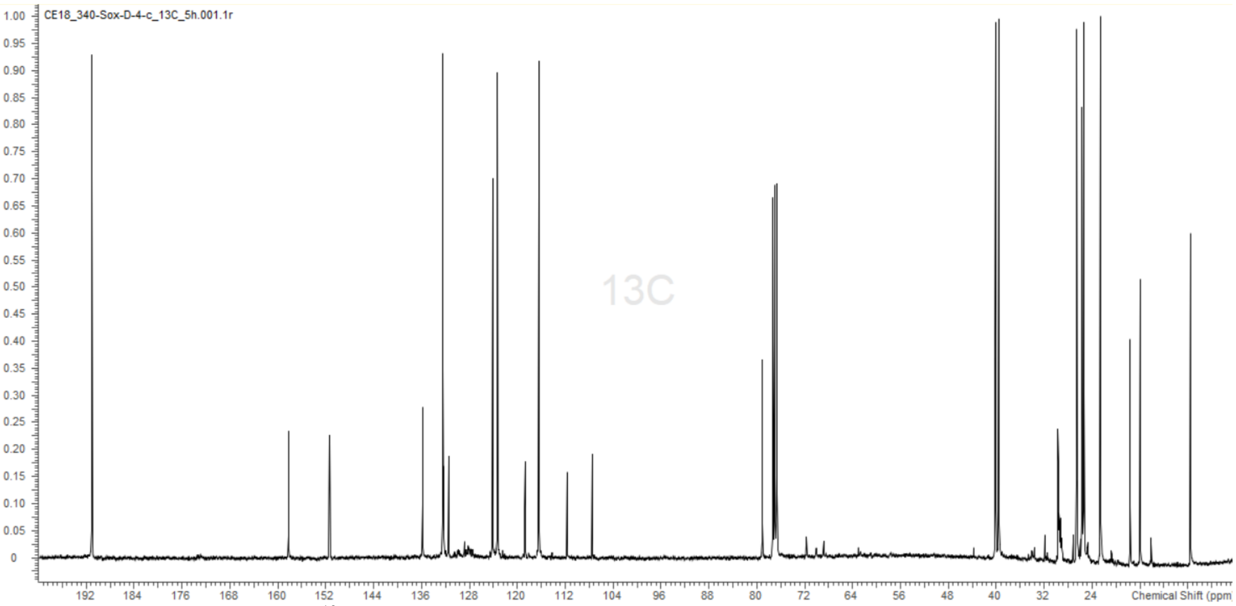

Figure S3: Tuaimenal A (**1**) ^{13}C NMR spectrum (100 MHz, $CDCl_3$).

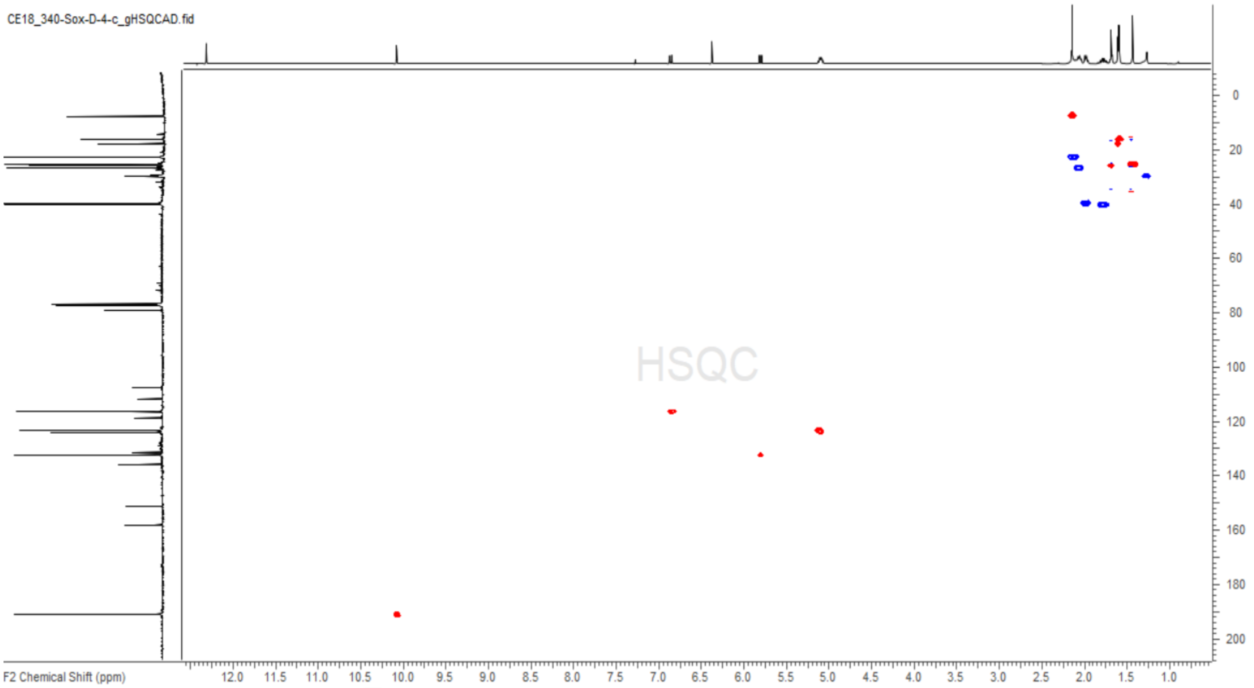

Figure S4: Tuaimenal A (**1**) HSQC spectrum (400 MHz, $CDCl_3$).

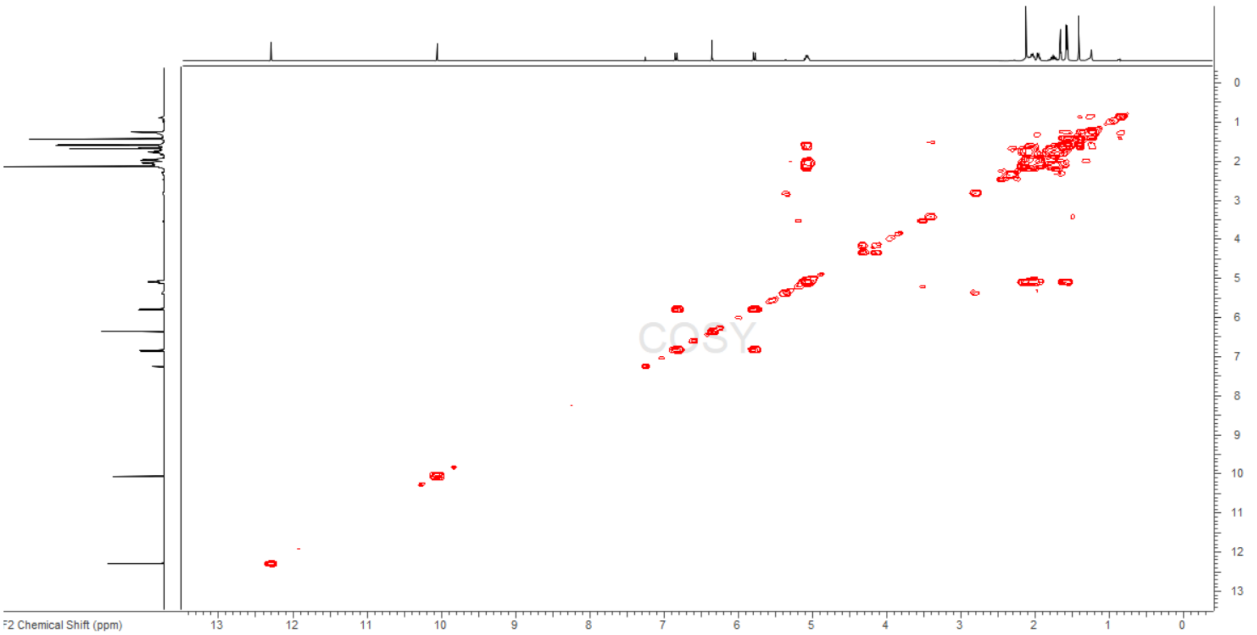

Figure S5: Tuaimenal A (**1**) gCOSY spectrum (400 MHz, $CDCl_3$).

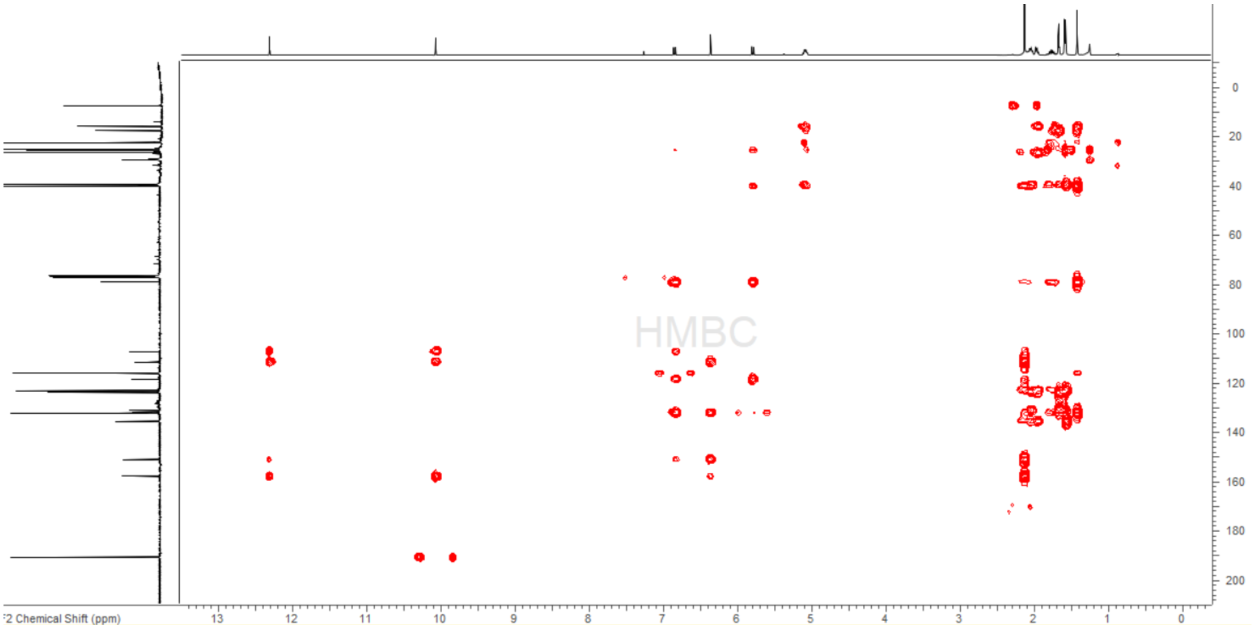

Figure S6: Tuaimenal A (**1**) HMBC spectrum (400 MHz, $CDCl_3$).

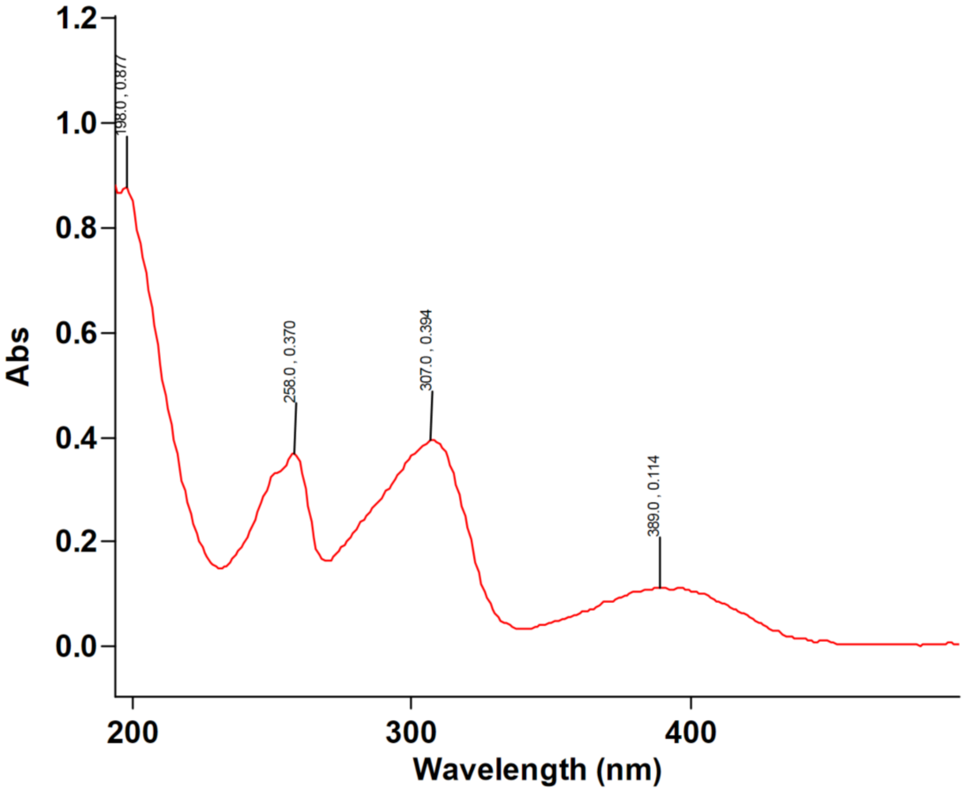

Figure S7: Tuaimenal A (**1**) UV spectrum in ACN (10 µg/mL); λ_{max} (log ε) 198 nm (3.2), with additional peaks noted at λ (log ε) 258 nm (1.4), 307 nm (1.5), and 369 nm (0.3).

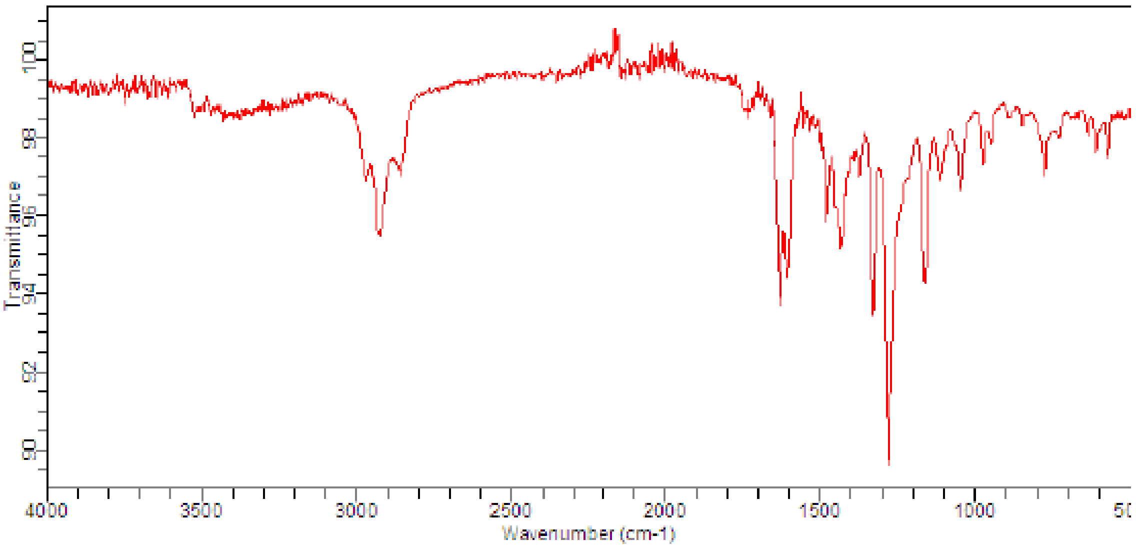

Figure S8: Tuaimenal A (**1**) FTIR spectrum, thin film.

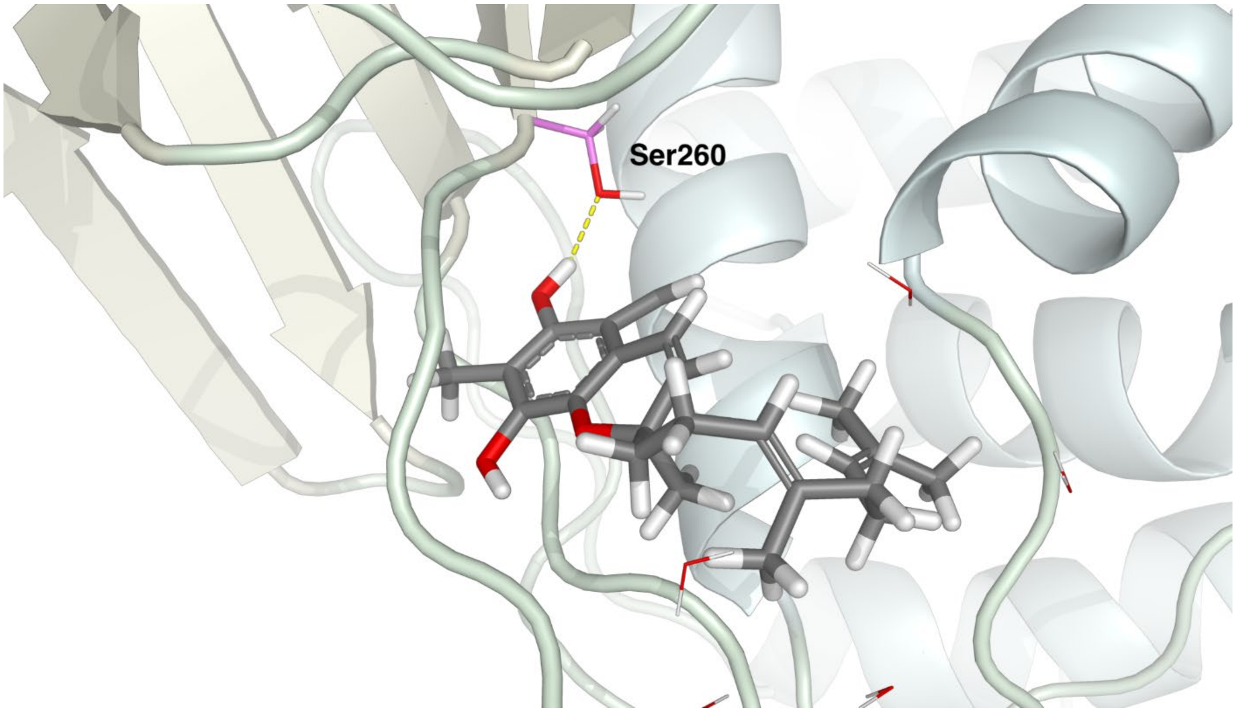

Figure S9: Final pose from the flexible docking of tuaimenal A (**1**) into PLpro. Dashed yellow lines indicate a hydrogen bond to Ser260.

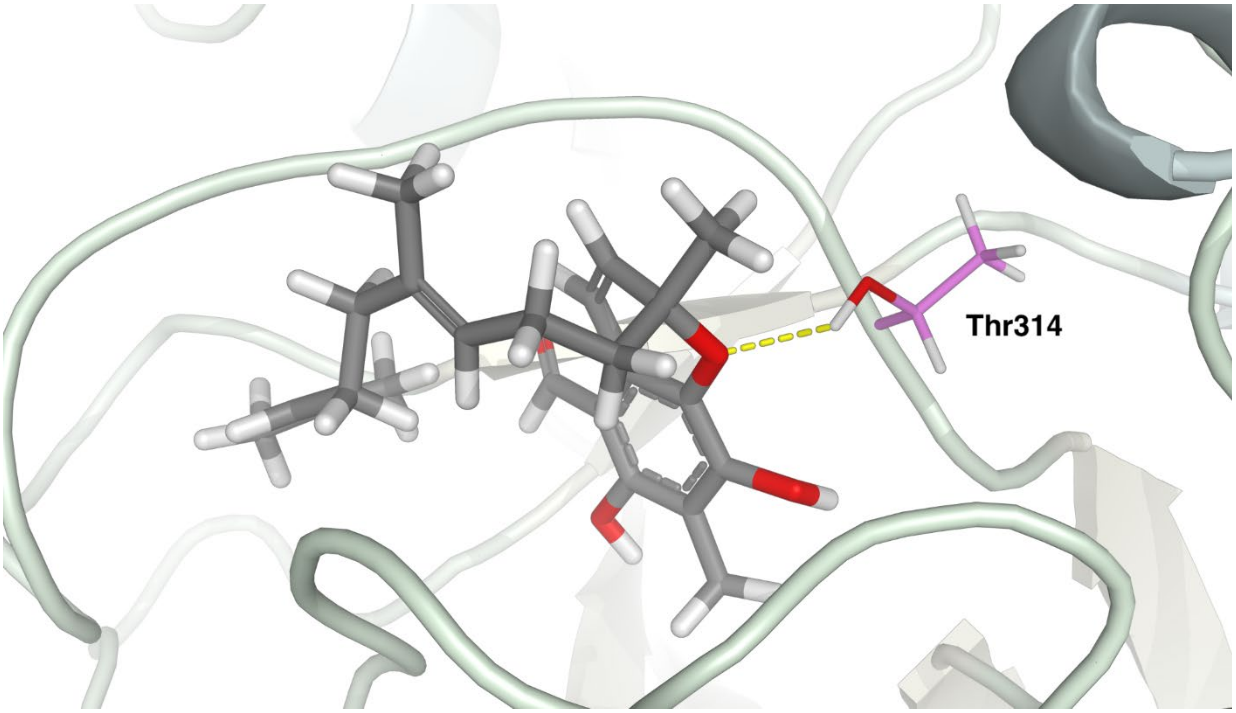

Figure S10: Final pose from the flexible docking of tuaimenal A (**1**) into TMPRSS2. Dashed yellow lines indicate a hydrogen bond to Thr314.

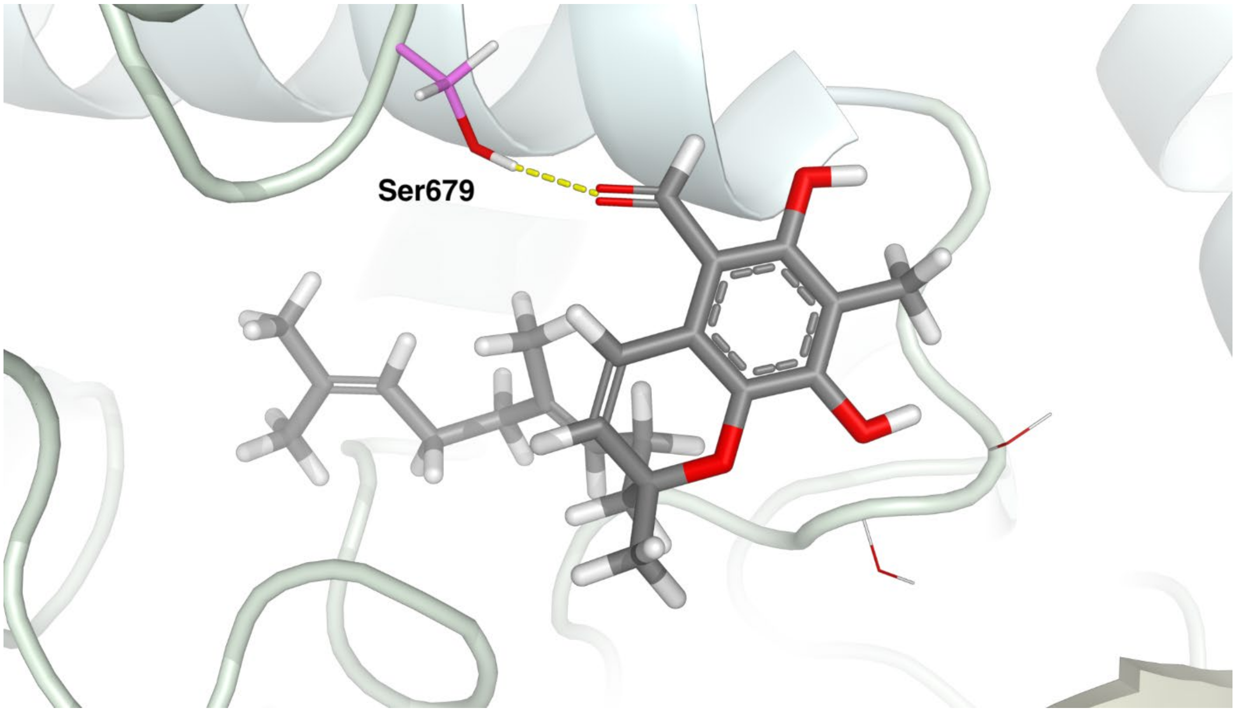

Figure S11: Final pose from the flexible docking of tuaimenal (**1**) A into RdRp. Dashed yellow lines indicate a hydrogen bond to Ser679.

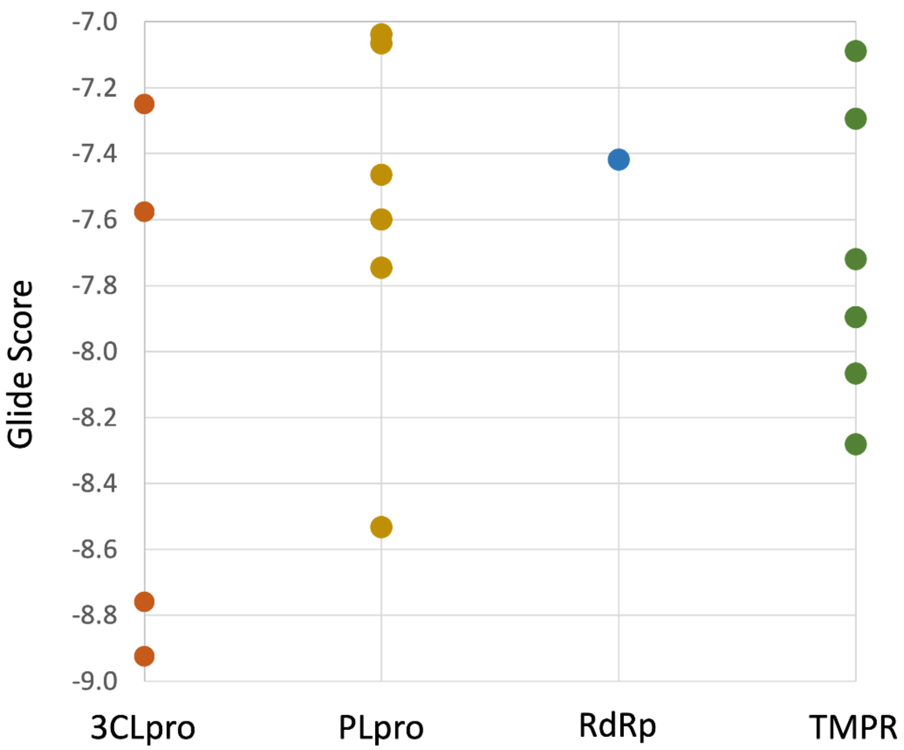

Figure S12. Glide scores for SARS-CoV-2 protein targets for the ligand conformations of tuaimenal A (**1**).

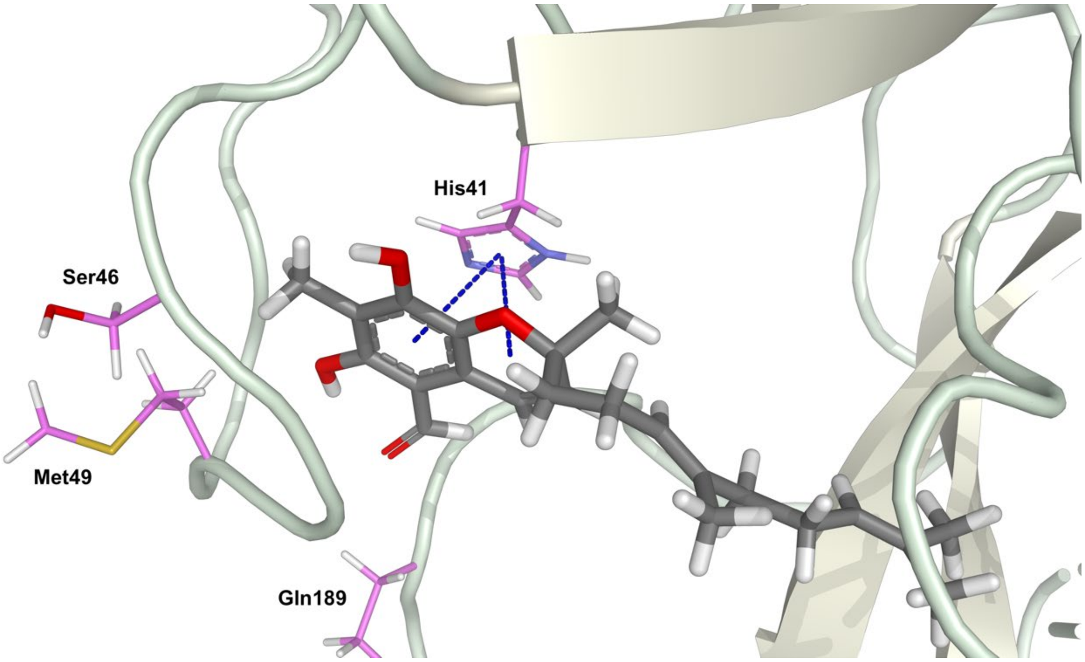

Figure S13. Final pose of tuaimenal A (**1**) Glide docking with 3CLpro, highlighting the two pi-pi stacking interactions (in dashed blue lines) with His41. Hydrogen bonds with Ser46, Met49, and Gln189 are to backbone atoms and not pictured.

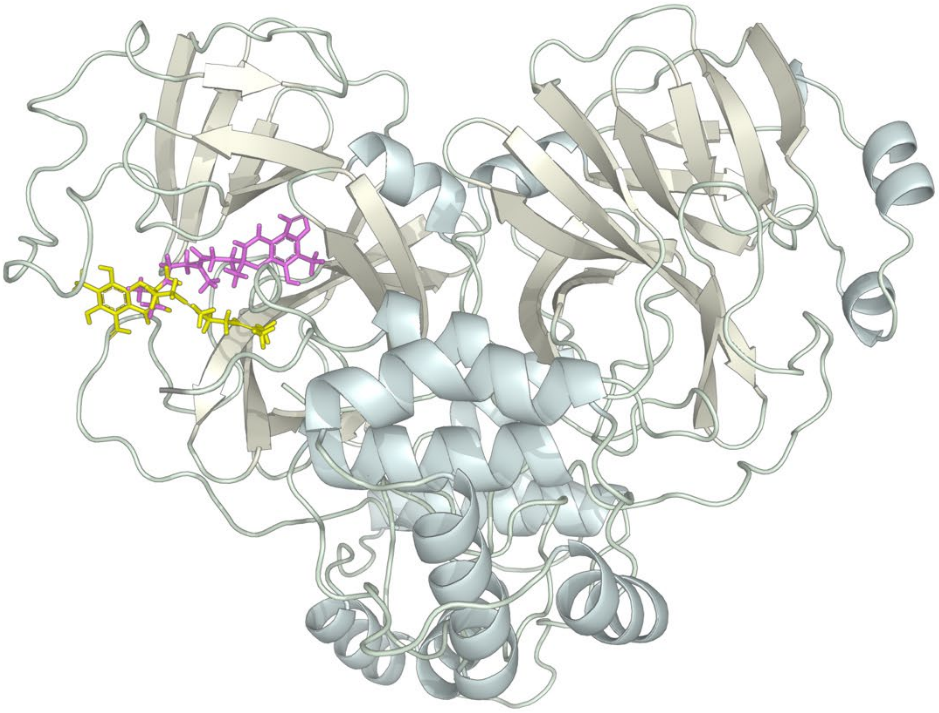

Figure S14. Structure of Mpro with the final ligand poses from rigid and flexible docking superimposed. The flexible docking pose, in purple, can be found deeper in the active site than that of the rigid docking pose, in yellow.

Rigid Docking Methods and Results. Schrödinger's Glide application was used to conduct the initial molecular docking of tuaimenal A (1). Ligand conformations were sampled flexibly while the protein receptor remained rigid. The van der Waals radii of the ligand atoms was set to a scaling factor of 0.80 and a partial atomic charge cutoff of 0.15 to soften the potential when considering nonpolar regions of the ligands. The prepared ligands were then computationally bound to the respective targets using extra precision (XP) virtual screening settings.

Validation of binding affinity is observed when multiple ligand conformations are bound to multiple target conformations of the same protein. Six favorable poses of tuaimenal A were observed across three of the 3CLpro structures (i.e., three different protein conformations of 3CLpro out of the five total initial receptors), of which the lowest Glide score was -8.925. Eleven favorable poses resulted for three of the PLpro conformations (lowest Glide score of -8.533, average -7.585). Seven favorable poses resulted for three human TMPRSS2 structures (lowest Glide score of -8.282, average -7.750). Only one conformation of tuaimenal A bound favorably to the viral RdRp structure (Glide score of -7.419).

Table S1: Information on the residue numbers selected in the definition of the active site of each protein target.

3CLpro	PLpro	RdRp	TMPRSS2
20	160	465	135
25	162	473	136
26	164	475	151
27	165	476	152
28	206	478	157
40	244	542	200
41	245	543	290
42	246	600	292
140	247	602	293
141	261	607	294
142	262	608	315
143	263	610	316
144	265	611	317
145	267	612	318
146	268	614	319
147	269	678	320
162	270	680	321
163	271	681	325
164	272	733	326
165		734	327
189			328
			329

Table S2:
Favorable Glide scores received by 6 poses of tuaimenal A (1) docked into 5 conformations of each protein target.

Protein target	Glide score
3CLpro	-8.925
3CLpro	-8.925
3CLpro	-8.760
PLpro	-8.533
PLpro	-8.533
TMPR	-8.282
TMPR	-8.067
TMPR	-7.896
TMPR	-7.896
PLpro	-7.747
PLpro	-7.747
TMPR	-7.721
PLpro	-7.600
PLpro	-7.600
3CLpro	-7.577
3CLpro	-7.577
PLpro	-7.465
RdRp	-7.419
TMPR	-7.295
3CLpro	-7.250
TMPR	-7.090
PLpro	-7.066
PLpro	-7.066
PLpro	-7.039
PLpro	-7.039

Title:

Absolute Configuration Determination Report

**GENERAL INFORMATION**	
**Customer**	USF / Nicole
**Sales Order Number**	2020-115 LSV
**Sample code (Our ref.)**	**Tuaimenal A**
**Sample description (Your ref.)**	**Tuaimenal A**
**VCD-spectrometer**	ChiralIR w/ Dual*PEM*
**Report prepared by**	Jordan Nafie
**Report validated and signed by**	Rina K Dukor
**Date**	February 12, 2021
**RESULTS**	
Absolute Configuration of Tuaimenal A is (R)	Confidence Level: **86%**
**MEASUREMENT PARAMETERS**	
**Concentration**	5.7mg / 220uL
**Solvent**	$CDCl_3$
**Resolution**	4 cm^{-1}
**PEM setting**	1400 cm^{-1}
**Number of scans/Measurement time**	24 hours
**Sample cell**	BaF_2
**Path length**	100 μm
**CALCULATION DETAILS**	
**Gaussian version**	Gaussian 09
**Total low-energy conformers used for Boltzmann sum**	338
**Methodology and basis set for DFT calculations**	B3LYP/6-31Gd w/CPCM (chloroform)
**Enantiomer used for calculation**	R
**Total calculated conformers**	818
**Number of low-energy conformations shown in report**	4

COMMENTS

The confidence level is a measure of the degree of congruence between a calculated and measured spectrum. If identical spectra are being compared the confidence level is 100%. The confidence level (CL) is not the likelihood that the assignment is correct. Rather it's a measure of quality or degree of agreement between calculated and measured spectra. With a CL of 86% for this molecule, the visual agreement between measured and calculated spectra is good – this is a high confidence assignment. Due to the flexibility and high number of active conformers, the VCD intensity for this molecule was very low. Despite some noise in the measured spectrum, the results clearly point to an R configuration.

17546 Bee Line Hwy | Jupiter, Florida 33458, USA
ph. 561 625.0133 | fax. 561 625.0717 | www.btools.com

Title:

Absolute Configuration Determination Report

Structure of Tuaimenal A:

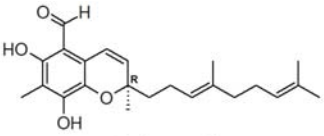

Tuaimenal A

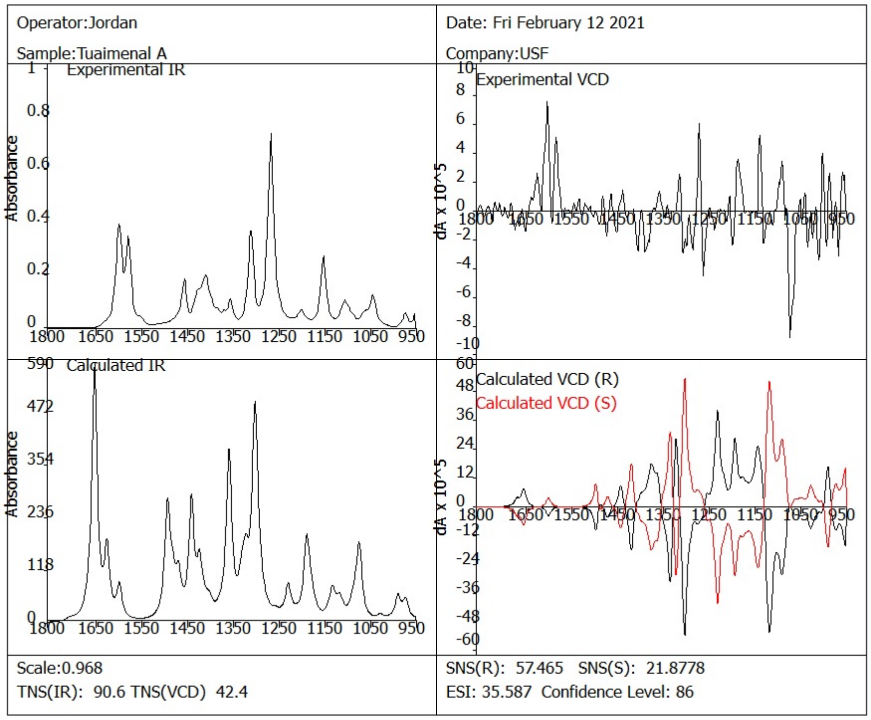

CompareVOA Results.
Please note: In this plot the frequency scaling factor is not applied.

Absolute Configuration Determination Report

Table 1. Numerical comparison describing the similarity in the range of 950 - 1800 cm^{-1} between the calculated IR and VCD spectra for the **(R)** enantiomer at the B3LYP / 6-31Gd w/ CPCM (Chloroform) level and the observed IR and VCD spectra for **Tuaimenal A**.

Cal. (950-1800cm^{-1})	Numerical comparison	Observed **Tuaimenal A**
**(R)**	scaling factor	0.968
	IR similarity (%)	90.6
	[a]∑ (%)	57.465
	[b]Δ (%)	35.587
	Confidence Level (%)	86

[a]∑: single VCD similarity, gives the similarity between the calculated and observed VCD spectra.
[b]Δ: enatiomeric similarity index, gives the difference between the values of ∑ for both enantiomers of a given diastereoisomer.

17546 Bee Line Hwy │ Jupiter, Florida 33458, USA
ph. 561 625.0133 │ fax. 561 625.0717 │ www.btools.com

Title:

Absolute Configuration Determination Report

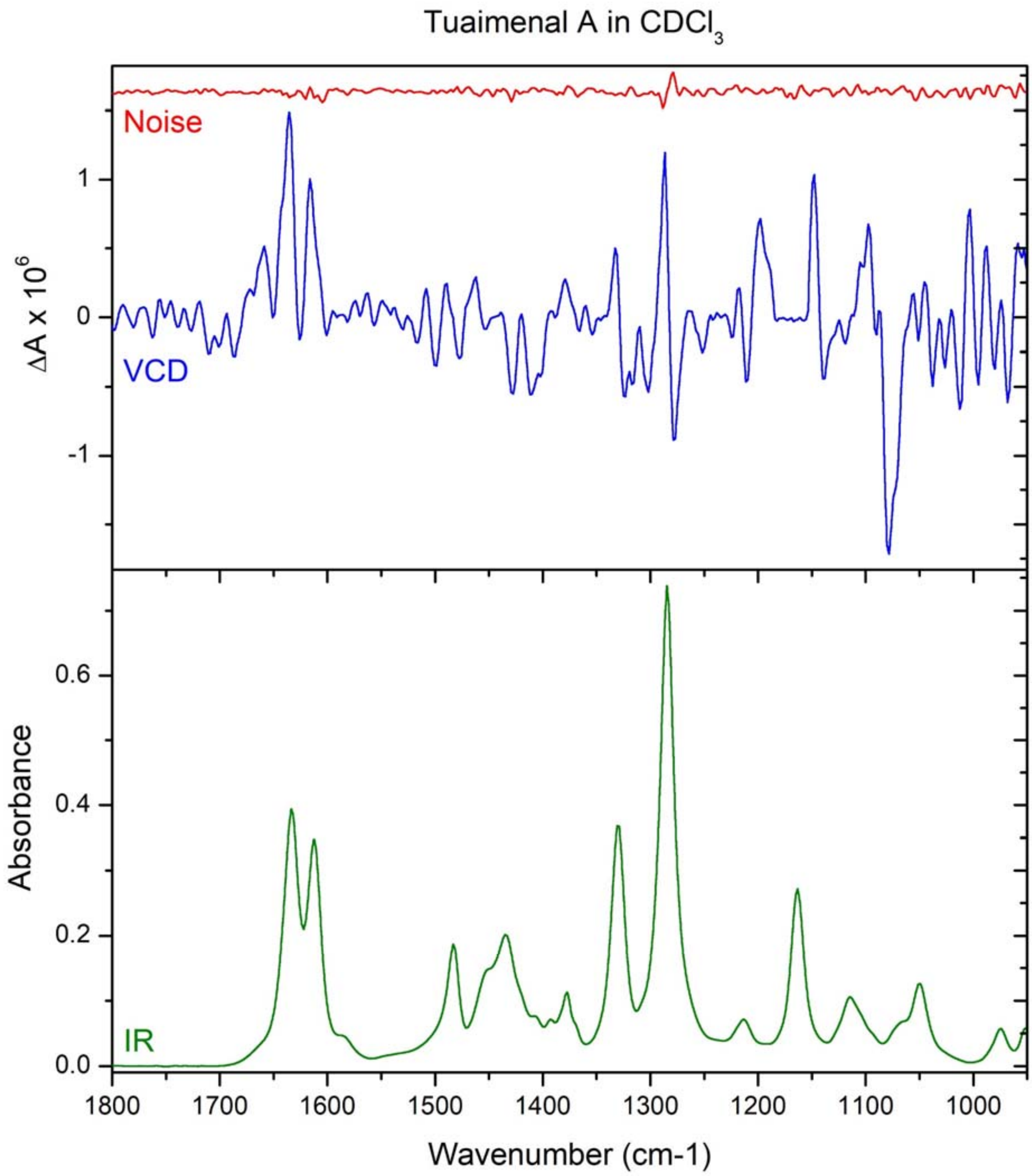

IR (lower frame) and VCD (upper frame) spectra of **Tuaimenal A** in $CDCl_3$; 100um path-length cell with BaF_2 windows; 24 h collection for enantiomer and solvent; instrument optimized at 1400 cm^{-1}. Solvent subtracted IR and VCD spectra are shown. Uppermost trace is the VCD noise spectrum.

17546 Bee Line Hwy │ Jupiter, Florida 33458, USA
ph. 561 625.0133 │ fax. 561 625.0717 │ www.btools.com

Title:

Absolute Configuration Determination Report

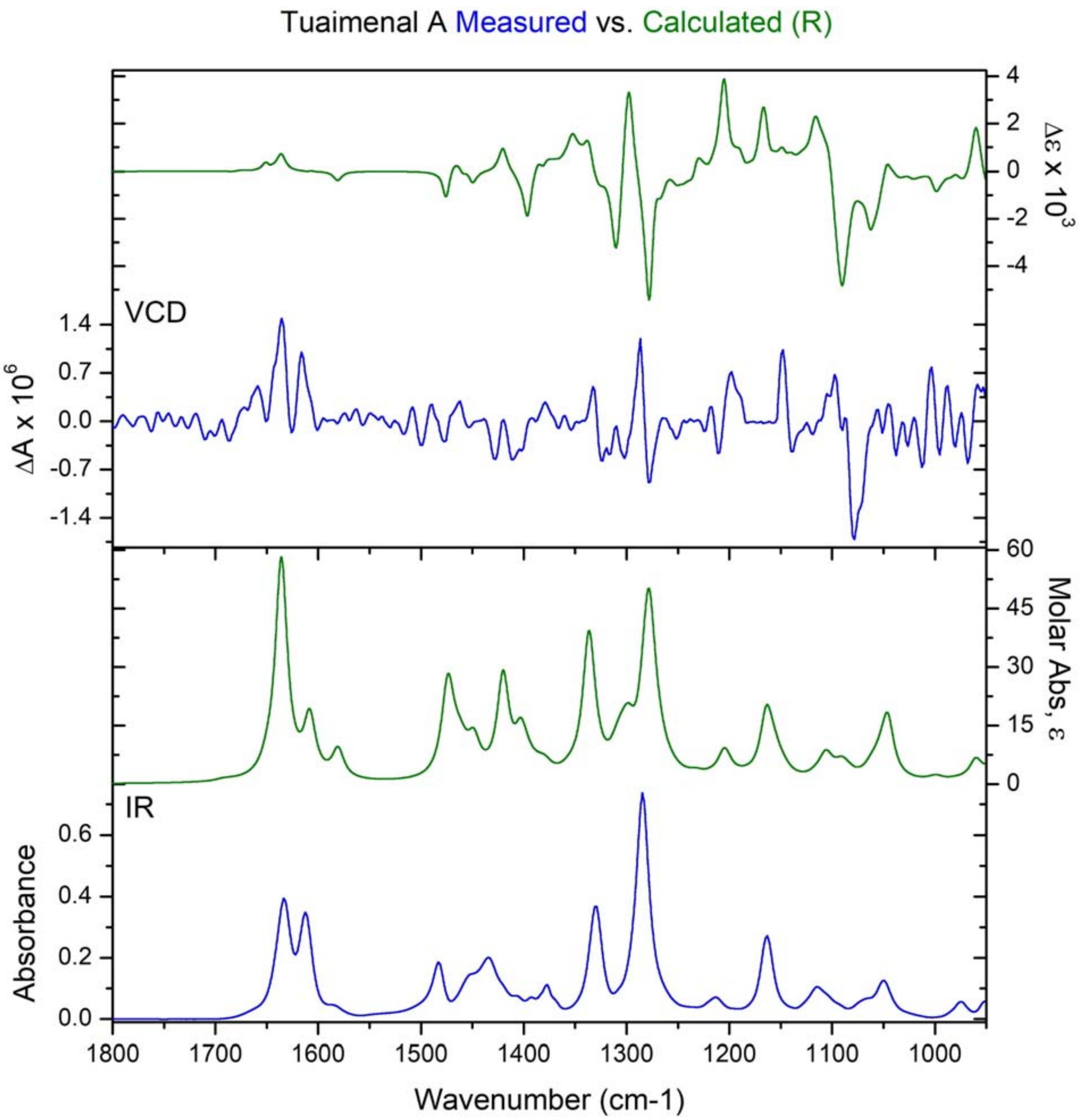

IR (lower frame) and VCD (upper frame) spectra observed for **Tuaimenal A** (left axes) compared with Boltzmann-averaged spectra of the calculated conformations for the **(R)** configuration, (right axes).

17546 Bee Line Hwy │ Jupiter, Florida 33458, USA
ph. 561 625.0133 │ fax. 561 625.0717 │ www.btools.com

Absolute Configuration Determination Report

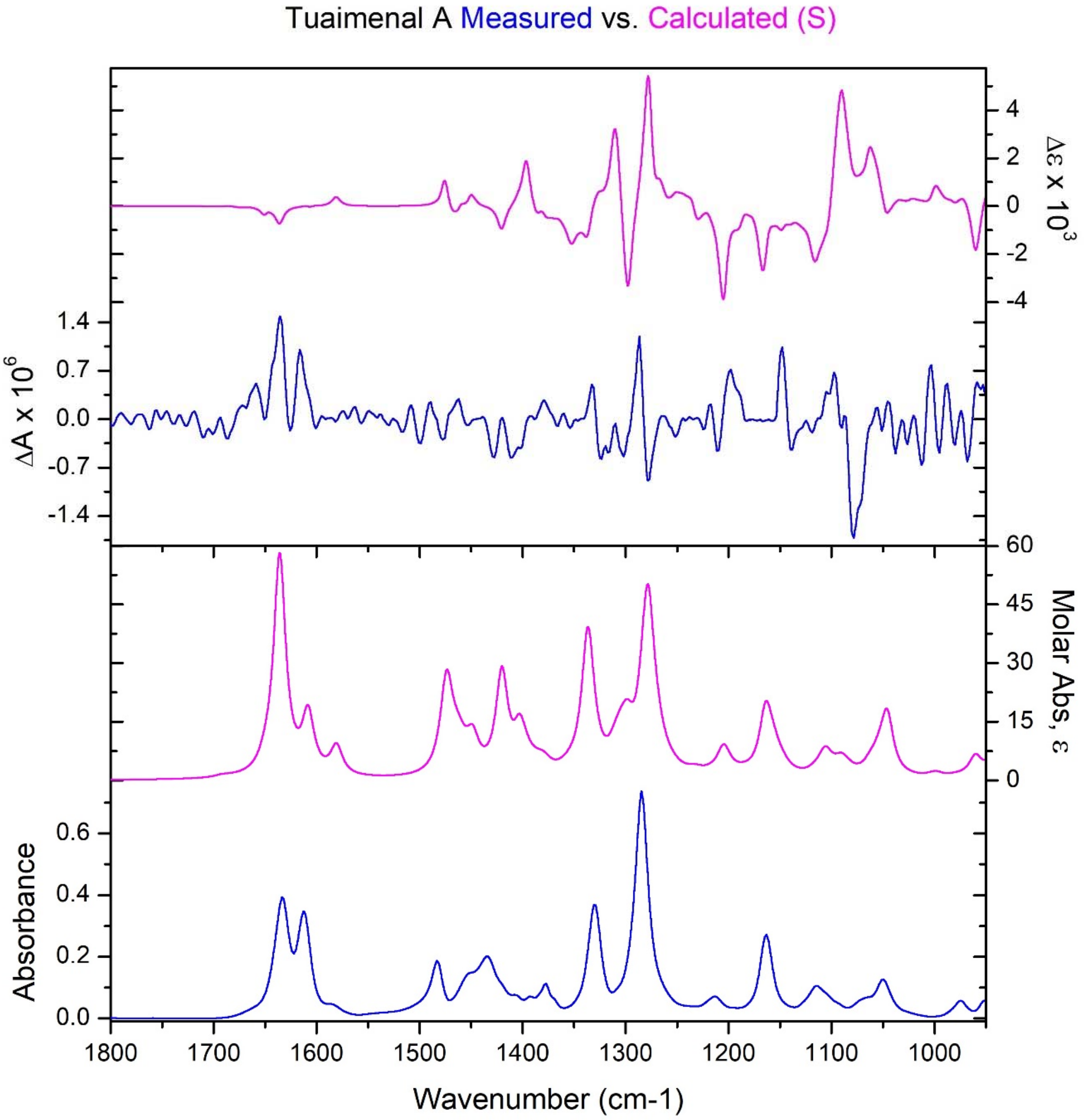

IR (lower frame) and VCD (upper frame) spectra observed for **Tuaimenal A** (left axes) compared with Boltzmann-averaged spectra of the calculated conformations for the **(S)** configuration, (right axes).

Absolute Configuration Determination Report

Lowest energy conformers - (R) Configuration:

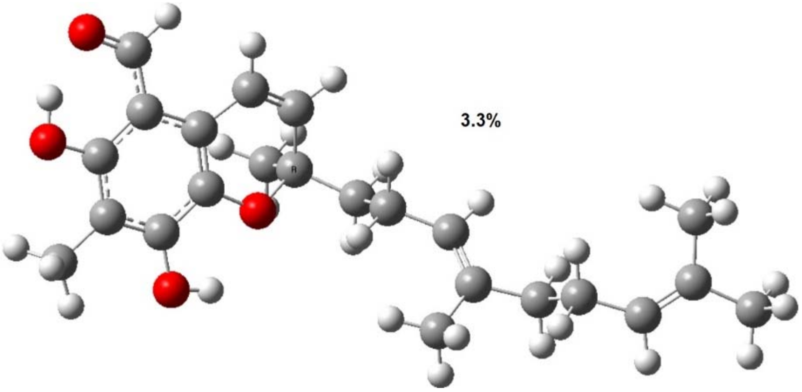

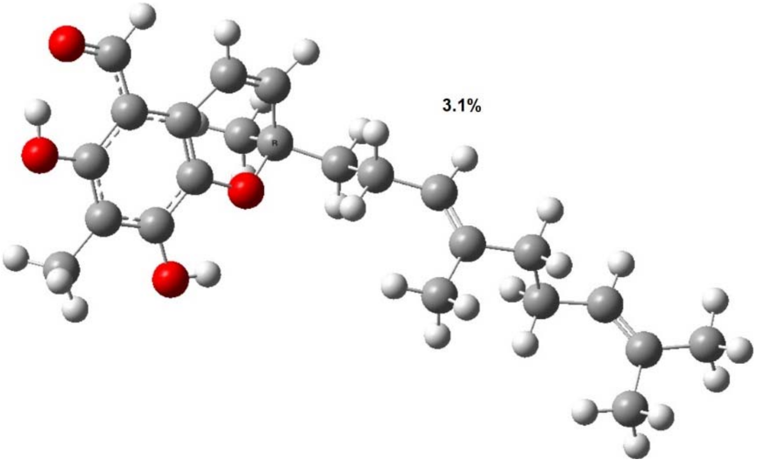

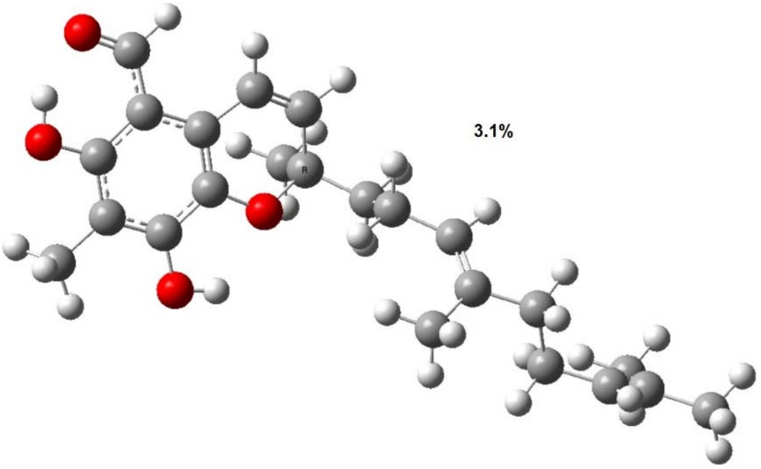

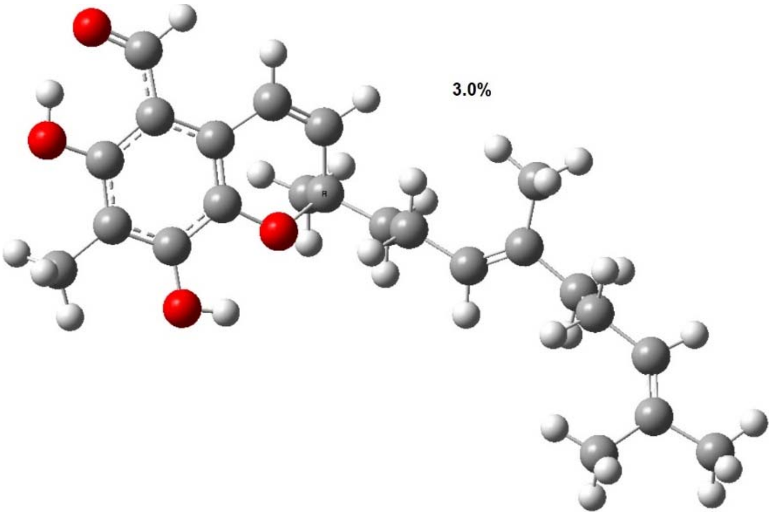

Absolute Configuration Determination Report

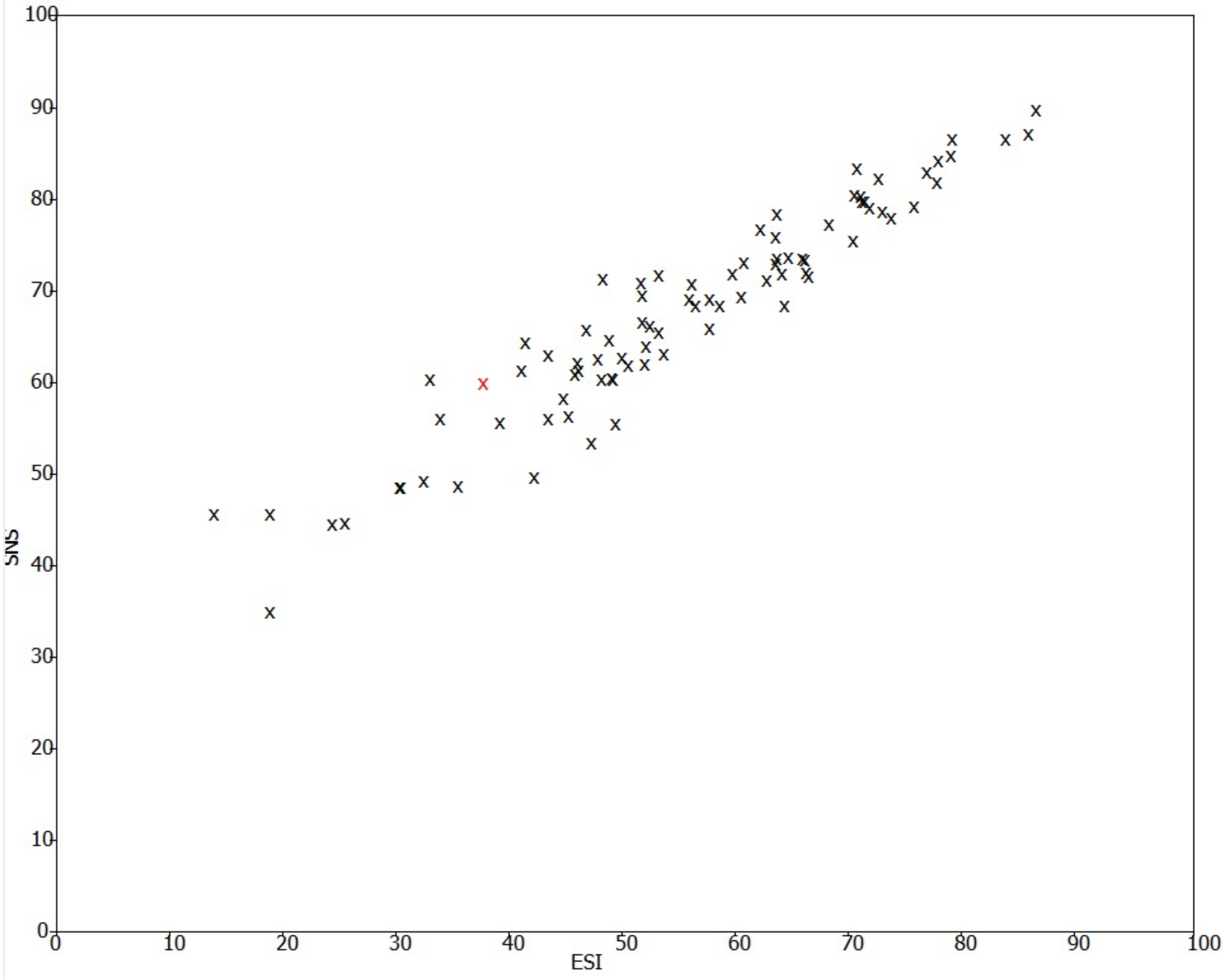

Plot of ESI (similarity of correct enantiomer minus incorrect enantiomer to calculated) vs SNS (overall similarity of correct enantiomer to calculated) for a library of correct assignments verified independently by X-Ray other method (Black X marks). Red X is **Tuaimenal A**.

17546 Bee Line Hwy | Jupiter, Florida 33458, USA
ph. 561 625.0133 | fax. 561 625.0717 | www.btools.com
